# *CADEE*: Computer-Aided Directed Evolution of Enzymes

**DOI:** 10.1107/S2052252516018017

**Published:** 2017-01-01

**Authors:** Beat Anton Amrein, Fabian Steffen-Munsberg, Ireneusz Szeler, Miha Purg, Yashraj Kulkarni, Shina Caroline Lynn Kamerlin

**Affiliations:** a Science for Life Laboratory, Department of Cell and Molecular Biology, Uppsala University, BMC Box 596, S-751 24 Uppsala, Sweden

**Keywords:** computational directed evolution, computational enzyme design, distributed computing, empirical valence bond, triosephosphate isomerase

## Abstract

The tremendous interest in enzymes as biocatalysts has led to extensive work in enzyme engineering, as well as associated methodology development. Here, a new framework for computer-aided directed evolution of enzymes (*CADEE*) is presented which allows a drastic reduction in the time necessary to prepare and analyze *in silico* semi-automated directed evolution of enzymes. A pedagogical example of the application of *CADEE* to a real biological system is also presented in order to illustrate the *CADEE* workflow.

## Abstract 2

A new computational tool, *CADEE* (Computer-Aided Directed Evolution of Enzymes), is presented.

## 1. Introduction   

In recent years there has been an explosion of interest in enzymes as biocatalysts for a wide range of processes from chemical synthesis to generating new biofuels (Reetz, 2013 ▸; Nestl *et al.*, 2014 ▸; Bommarius, 2015 ▸; Faber *et al.*, 2015 ▸; Zhang, 2015 ▸). In particular, the excellent chemoselectivity, regioselectivity and enantioselectivity of enzymes, as well as their ability to work under mild reaction conditions, are the main factors that make enzymes competitive catalysts even on an industrial scale (Huisman & Collier, 2013 ▸). However, the same features that make enzymes such powerful catalysts *in vivo* can be contrary to the needs of industrial catalysts. For example, the majority of natural proteins have evolved to perform at the low substrate concentrations and catalyst loads found in physiological conditions; in contrast, economically feasible chemical processes require high loads to be able to achieve reasonable space–time yields (Tufvesson *et al.*, 2013 ▸). Fortunately, recent advances in protein-engineering approaches have provided great scope for enzyme adaptation to match process requirements, rather than tuning processes to the limitations of the catalyst, as was performed as recently as ten to fifteen years ago (Bornscheuer *et al.*, 2012 ▸). As a result of this, the number of industrially applicable enzymes available is steadily increasing (Huisman & Collier, 2013 ▸; Choi *et al.*, 2015 ▸; Narancic *et al.*, 2015 ▸). However, despite these advances, there still remain many open challenges in the field (some of which are reviewed in detail in, for example, Bommarius, 2015 ▸), and computation in particular has been increasingly employed as a tool to accelerate progress in enzyme-(re)design efforts (Kiss *et al.*, 2013 ▸; Kries *et al.*, 2013 ▸; Damborský & Brezovský, 2014 ▸; Frushicheva *et al.*, 2014 ▸; Świderek, Tuñón, Moliner *et al.*, 2015 ▸).

Historically, protein-engineering efforts have focused on rational design approaches, which have made significant contributions to the field (for reviews, see, for example, Steiner & Schwab, 2012 ▸; Tiwari *et al.*, 2012 ▸). These are, however, limited by the complexity of enzymes and the large amount of information required to make reliable predictions. Although effort in rational design still continues, the achievements of these methods are often limited and require further optimization (Steiner & Schwab, 2012 ▸; Tiwari *et al.*, 2012 ▸; Kiss *et al.*, 2013 ▸; Kries *et al.*, 2013 ▸, Frushicheva *et al.*, 2014 ▸). This optimization is typically achieved by the use of methods that have literally revolutionized biocatalysis, namely strategies that allow the guided laboratory evolution of enzymes (so-called ‘directed evolution’ approaches). These involve various gene diversification and screening or selection strategies (Packer & Liu, 2015 ▸). Directed evolution is a particularly powerful tool for biocatalysis, as smart combinations of diversification methods and screening strategies increasingly allow the production of catalytically superior enzyme variants that are simply not predictable through rational design approaches (Arnold & Volkov, 1999 ▸; Jäckel *et al.*, 2008 ▸; Currin *et al.*, 2015 ▸).

The greatest challenge facing directed evolution approaches is the sheer vastness of the sequence space that needs screening. That is, even a simple 300-amino-acid protein can have 300^20^ possible permutations of amino-acid substitutions. For comparison, traditional microtitre plate screens are typically limited to a throughput of ∼10^4^ clones per round (Packer & Liu, 2015 ▸). Here, there have fortunately been significant advances in both screening and selection methods involving fluorescence-activated sorting (FACS; *e.g.* cell surface display or *in vitro* compartmentalization), which make it now possible to achieve throughput limits of 10^8^–10^10^ (Packer & Liu, 2015 ▸). However, even though advanced screening and selection methods have substantially decreased the screening effort, the sequence space of most enzymes is still far too large to be sufficiently covered by directed evolution, making such studies something of a ‘shot in the dark’. Additionally, depending on the starting points used, directed evolution experiments can easily become stuck at local minima in sequence space (Romero & Arnold, 2009 ▸; Gumulya *et al.*, 2012 ▸).

**Figure 1:**
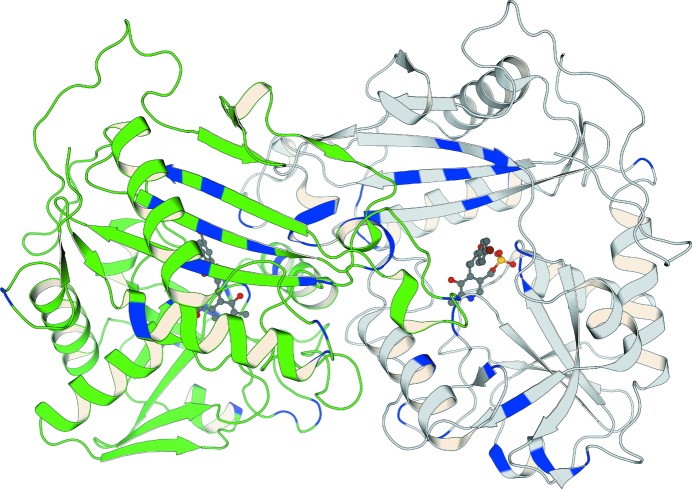
A prominent example of semi-rational directed evolution of an (*R*)-selective amine transaminase for sitagliptin manufacture (Savile *et al.*, 2010 ▸). Shown here is the structure of the final variant after 11 rounds of evolution (PDB entry 5fr9; Cuetos *et al.*, 2016 ▸). Achieving this industrially applicable enzyme required 27 amino-acid substitutions, the positions of which are highlighted here in blue in both chains of the structure. This figure illustrates how diversely distributed across an enzyme functionally important residues can be, and therefore why it can be so hard to predict appropriate amino-acid substitutions using only rational design approaches. Chain *A* is coloured light grey and chain *B* green, and the covalent cofactor–inhibitor complex of both subunits is shown in dark grey using a ball-and-stick representation for better clarity. This and all figures showing crystal structures were created using the *PyMOL* Molecular Graphics System (Version 1.8, Schrödinger LLC).

To finally achieve more broadly applicable and practicable protein-engineering strategies, directed evolution needs to be combined with computational tools and/or structural information (Davids *et al.*, 2013 ▸). By creating ‘small but smart’ libraries, these semi-rational approaches have led to impressively redesigned enzymes for industrial approaches by reducing the screening efforts involved to economically feasible levels (Bornscheuer *et al.*, 2012 ▸). A prominent example of this is the case of an amine transaminase being re-engineered through 11 rounds of semi-rational directed evolution to match the needs of the industrial process for sitagliptin manufacture (Savile *et al.*, 2010 ▸). This was achieved by first predicting positions for saturation mutagenesis to improve the substrate scope using homology modelling. Once a feasibly high activity had been achieved towards the target substrate, random substitutions were introduced that were then recombined based on statistically analyzed sequence–activity relationships (ProSAR; Fox *et al.*, 2007 ▸). Through this support from rational methods, it was possible to substantially reduce the amount of screening effort required for the directed evolution to be successful. The final variant, which bears 27 substitutions (Fig. 1 ▸), has impressively improved tailored catalytic properties, and eventually replaced the rhodium-catalyzed amination step in the classical process for sitagliptin manufacture.

In addition, computational modelling and simulations have demonstrated themselves to be increasingly powerful tools in computational enzyme design. The contributions of theory range from the *de novo* design of enzymes with novel catalytic properties (Kiss *et al.*, 2013 ▸; Kries *et al.*, 2013 ▸), through structural bioinformatics and machine-learning tools for hotspot prediction (Fox *et al.*, 2007 ▸; Bendl *et al.*, 2016 ▸) and attempts towards *in silico* directed evolution (Verma *et al.*, 2012 ▸), to the use of molecular-dynamics simulations and quantum-mechanical calculations to partially rationalize the design process (Privett *et al.*, 2012 ▸; Jiménez-Osés *et al.*, 2014 ▸; Noey *et al.*, 2015 ▸; Osuna *et al.*, 2015 ▸; Wijma *et al.*, 2015 ▸; Romero-Rivera *et al.*, 2016 ▸). These examples demonstrate the role of theory by drastically reducing the screening effort required in directed evolution studies, and thus maximizing the likelihood of the successful engineering of enzymes. Furthermore, theory can make valuable contributions by providing insight into why some evolutionary trajectories hit functional dead-ends on which further optimization is not possible (Voigt *et al.*, 2000 ▸; Privett *et al.*, 2012 ▸; Hallen *et al.*, 2013 ▸). The accumulated knowledge can then be applied to guide future directed evolution experiments and to pinpoint properties that are not observable by experiments alone.

While several possible theoretical approaches exist that one could use as a baseline with which to perform *in silico* directed evolution experiments (Verma *et al.*, 2012 ▸), recent studies have highlighted the power of the empirical valence-bond (EVB) approach in particular as an important tool in the computational design of new enzymes (Roca *et al.*, 2009 ▸; Frushicheva *et al.*, 2010 ▸; Fuxreiter & Mones, 2014 ▸). This approach is a valence-bond (VB)-based classical approach that uses VB theory to describe chemical reactivity in a quantum-mechanical framework and, as it is inherently a classical approach, it carries the advantage of being sufficiently fast to simulate the large numbers of amino-acid substitutions necessary for *in silico* screening. At the same time, as this approach uses rigorously parameterized classical force fields, it provides a tremendous amount of physical information and thus allows the modelling of bond-making and bond-breaking processes in a physically meaningful way. This makes EVB in particular powerful for performing *in silico* directed evolution, and we therefore present here our new toolbox *CADEE*, a simulation package/framework that allows (mostly) automated computer-aided directed evolution of enzymes. We note that while other approaches that allow *in silico* directed evolution already exist, most are focused mainly on predicting mutation hotspots using sequence information rather than quantitatively assessing the effect of different amino-acid substitutions on the corresponding activation free energies for the chemical step of catalysis (Verma *et al.*, 2012 ▸; Damborský & Brezovský, 2014 ▸). In addition, there have been elegant studies that demonstrate the possibility of screening mutational effects on the chemistry *in silico* using computational approaches (Hediger *et al.*, 2012 ▸; Wijma *et al.*, 2014 ▸); however, owing to the higher computational cost associated with these approaches, they have been limited to at best several hundred amino-acid substitutions, whereas, as we demonstrate here, *CADEE* can screen the quantitative effect of at least several thousand amino-acid substitutions with reasonable computational cost (as well as being easily scaled according to the computational resources available).

We have implemented *CADEE* as a framework around a specially modified version of the *Q* simulation package (Marelius *et al.*, 1998 ▸). The fact that *Q* is used as our main simulation engine allows our framework to exploit the full functional capabilities of this simulation package, as well as providing accessibility to a broad range of force fields and solvent models (see http://www.icm.uu.se/cbbi/aqvist-lab/q for further details). In addition, the initial search time can in principle be radically reduced by combining *CADEE* with pre-screening using a range of structural bioinformatics approaches that allow the prediction of mutation hotspots, which can then be targeted for subsequent *in silico* mutagenesis using *CADEE*.

Finally, we also present an application of the EVB approach to a model system, specifically a proton-transfer reaction catalyzed by the enzyme triosephosphate isomerase (TIM; EC 5.3.1.1). This enzyme is found in nearly every organism, as it is essential for glycolysis (Wierenga *et al.*, 2010 ▸). We chose this system as our showcase both based on the extensive experimental data available from studies on multiple organisms (see, for example, among many other studies, Straus *et al.*, 1985 ▸; Nickbarg *et al.*, 1988 ▸; Blacklow & Knowles, 1990 ▸; Sampson & Knowles, 1992 ▸; Malabanan *et al.*, 2011 ▸; Zhai *et al.*, 2015 ▸; Richard *et al.*, 2016 ▸) and also because of the chemical simplicity of the process that we are modelling, which in turn reduces the corresponding computational complexity. We emphasize, however, that our purpose in this study is *not* to redesign TIM, as this is already a very proficient enzyme (Albery & Knowles, 1976 ▸), but rather to use it as a pedagogical example to illustrate the *CADEE* workflow owing to the simplicity of the reaction involved. That is, the ability of the EVB approach to reliably model and predict mutational (and other) effects in a range of biomolecular systems has been well established elsewhere (Warshel *et al.*, 2006 ▸; Frushicheva *et al.*, 2011 ▸), and therefore here we mainly want to illustrate the automation of our *in silico* directed evolution approach. Note, in addition, that usage of *CADEE* requires a well characterized and parameterized model system as a starting point, as well as extensive conformational sampling, and the accuracy of *CADEE* will therefore be limited by how rigorously the user has parameterized the system (as is the case with any simulation study). As the present case is only intended to serve as a pedagogical example, the timescales on which we have performed the simulations shown here could be too short to be able to reliably redesign TIM or any other enzyme, in particular when it comes to larger structural changes; however, they do demonstrate how *CADEE* can be used, as well as providing, by extrapolation, benchmarks of the computational resources that would be required to perform effective screening on a ‘real-world’ system. Overall, we believe that *CADEE* fills a niche in computational enzyme-design studies, as it allows quantitative guided directed evolution studies, based on a rational understanding of the systems involved, importantly taking into account the electrostatic environment, while providing the ability to rank the proposed constructs on the basis of predicted energetics.

## 2. Experimental   

### 2.1. The empirical valence-bond approach   

In order to perform effective screening of predicted activation barriers for chemical reactions in a large number of enzyme active sites, it is crucial to use an approach that is on the one hand fast enough to be able to perform the computations in a cost-effective way, while not taking too hard a hit on the corresponding quantitative accuracy of the calculations. Here, clearly, while there have been very promising attempts at enzyme design using high-level quantum-mechanical approaches (Röthlisberger *et al.*, 2008 ▸; Hediger *et al.*, 2012 ▸; Kiss *et al.*, 2013 ▸; Pratter *et al.*, 2013 ▸), these approaches are far too computationally expensive for the screening of tens of thousands of enzyme variants. While one could, in principle, switch to semi-empirical QM/MM approaches (Cui & Elstner, 2014 ▸; Mlýnský *et al.*, 2014 ▸) instead, which would allow the screening of far larger numbers of substitutions, here one quickly faces problems with the limited accuracy of the available approaches (Cui & Elstner, 2014 ▸; Mlýnský *et al.*, 2014 ▸; Thiel, 2014 ▸). To obtain (in our opinion) the best balance between these two limitations, our underlying methodology of choice for *CADEE* is the empirical valence-bond (EVB) approach (Warshel & Weiss, 1980 ▸; Hwang *et al.*, 1988 ▸), although we note that this is mainly for computational convenience and the underlying philosophy of *CADEE* is easily extendable to any preferred computational approach with sufficiently high speed and accuracy to be able to perform the extensive sampling needed for efficient computational enzyme design.

In brief, the EVB approach is a classical approach based on force-field descriptions of different reaction states, which at the same time provides a quantum-mechanical description of chemical reactivity within a valence-bond framework (Warshel & Weiss, 1980 ▸; Hwang *et al.*, 1988 ▸). This allows EVB to harness both the speed of classical, force-field-based approaches, while at the same time carrying a tremendous amount of chemical and thermodynamic information, allowing a physically meaningful description of bond-making and bond-breaking processes. In practice, the EVB approach takes into account the resonance, or diabatic, states, which correspond to distinct valence-bond structures describing reactant, product and any intermediate states. The potential energy of any diabatic state (*H*_11_ and *H*_22_) is described as in (1) for the *i*th state, where *R* and *Q* represent the atomic coordinates and charges of the reacting atoms (‘solute’), respectively, and *r* and *q* represent the atomic coordinates and charges of the surrounding environment (‘solvent’), which is usually either vacuum, solvent molecules or protein:
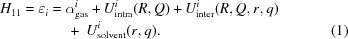



The first term in (1), α^*i*^_gas_, is the gas-phase energy of the *i*th diabatic state, while *U* ^*i*^_intra_(*R*, *Q*), *U* ^*i*^_inter_(*R*, *Q*, *r*, *q*) and *U* ^*i*^_solvent_(*r*, *q*) represent the intramolecular potential of the solute system (relative to its minimum), the interaction between the reacting atoms and the surrounding solvent atoms and the potential energy of the solvent, respectively. The off-diagonal elements *H*_*ij*_ can be described as exponential coupling functions of the distance between the reacting atoms (2),
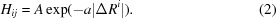



Finally, the adiabatic ground-state energy (*E*_g_), as well as its corresponding eigenvector (*C*_g_), are obtained from the lowest eigenvalue of the EVB Hamiltonian by solving the secular equation *H*_EVB_ *C*_g_ = *E*_g_ *C*_g_. The relevant activation free energies, Δ*G* ^‡^, can then be evaluated from this by adiabatically changing the system from one diabatic state to another. In the simplest case, which involves the two-state example discussed above, this can be achieved using a ‘mapping potential’, ∊_*m*_, of the form
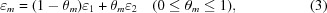
where θ_*m*_ is changed from 0 to 1 in *n* + 1 fixed increments θ_*m*_ = 0/*n*, 1/*n*, 2/*n*, …, *m*/*n*), with the system being forced to fluctuate near the transition state (TS) by means of potentials with one or more intermediate values of θ_*m*_. The free energy. Δ*G*_*m*_, associated with changing θ_*m*_ from 0 to *m*/*n* can then be simply evaluated using the well known free-energy perturbation/umbrella sampling (FEP/US) procedure, as outlined in detail in, for example, Warshel (1991 ▸) and Warshel *et al.* (2006 ▸). Finally, the free-energy functionals of the different diabatic states can be obtained by means of FEP/US using
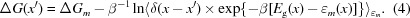



**Figure 2:**
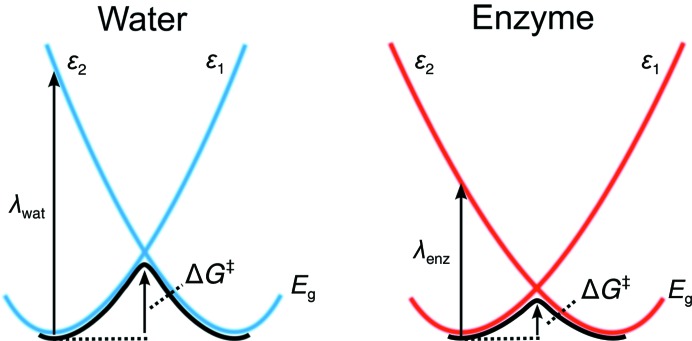
Schematic representation of the relationship between the different EVB diabatic states (∊_1_ and ∊_2_) in a simple two-state reaction, the corresponding adiabatic reorganization energy (λ) and the resulting activation barriers (Δ*G* ^‡^) in water (left) and in an enzyme (right). Both λ and Δ*G* ^‡^ are significantly smaller in the enzyme, *i.e.* the enzyme would be a catalyst for this hypothetical reaction (Warshel *et al.*, 2006 ▸).

Here, ∊_*m*_ is the mapping potential of (3) which keeps *x* in the region of *x*′. If the changes in ∊_*m*_ are sufficiently gradual, this will result in a free-energy functional, Δ*G*(*x*′), which is obtained with several values of *m* overlapping over a range of *x*′. Connecting the full set of Δ*G*_*i*_(*x*′) will yield the complete free-energy curve for the reaction (note that a similar construct can also be used to obtain the free-energy functional for each individual diabatic state; see Warshel, 1991 ▸). The origin of the catalytic effect can then be related to the EVB result by approximating the activation free energy using the modified Marcus equation (Warshel, 1991 ▸; Liu & Warshel, 2007 ▸),
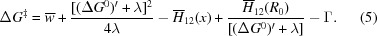



Here, 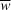 is the so-called ‘work term’ describing the free energy of bringing the reactant pair to the interaction distance, *R*_0_, at the reactant state. Δ*G* ^0^ corresponds to the reaction free energy, λ to the reorganization energy and Γ to the nuclear quantum-mechanical correction. 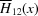 and 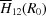 correspond to the average values of *H*_12_ at the transition and reactant states, respectively. Finally, 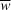 is related to the potential of mean force (PMF) of bringing the reacting groups together, and when this PMF is close to zero the work term is similar to the effect of bringing the reacting groups into a reacting ‘cage’, which is discussed in detail by, for example, Warshel *et al.* (2006 ▸). Note that λ can also be directly obtained from the EVB diabatic free energies, and it can also be estimated (Kamerlin & Warshel, 2010 ▸) by using the relationship
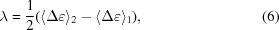
where 〈Δ∊〉 denotes the average difference between ∊_1_ and ∊_2_ from trajectories using either the potential of ∊_1_ or ∊_2_. In any case, the relationship between the diabatic states ∊_1_ and ∊_2_, ­the activation energy *E*_g_ and the reorganization energy λ in solution and in an enzyme active site can be defined schematically as shown in Fig. 2 ▸, from which it can be seen that both Δ*G* ^‡^ and λ are expected to be significantly smaller in the enzyme environment than for the corresponding reaction in solution.

As the adiabatic free-energy surface obtained using the EVB approach is dependent on the position of the two VB parabolas relative to each other (which is determined by the EVB parameters α and *H*_*ij*_ as described above), it is therefore necessary in an EVB framework to first identify an appropriate reference state to which to fit the EVB parabola. Once calibrated, these EVB parameters can then be used (unchanged) when moving from the reference state to other reaction environments, such as the active site of an enzyme or other catalyst, in order to capture the effect of changing the environment on the calculated energetics. Here, the appropriate reference state can be, for example, either the corresponding uncatalyzed reaction in aqueous solution (if appropriate experimental or computational information is available about the energetics of this process) or, alternately, if the mechanism of the reaction is already understood, it can also for instance be the energetics of the reaction in the wild-type (WT) enzyme relative to a series of mutants. In the present study, the reaction of interest is a simple proton-transfer reaction, the uncatalyzed energetics of which can be easily extrapolated from a combination of experimental studies on analogous systems and by simple p*K*_a_ considerations (see §[Sec sec2.4.2]2.4.2; Richard, 1984 ▸; Åqvist & Fothergill, 1996 ▸). The uncatalyzed solution reaction therefore provides an excellent benchmark against which to study the energetics of the corresponding reaction in the TIM active site, and this was therefore used as the reference reaction for the calibration of the EVB parameters used in this work (see §[Sec sec2.4.2]2.4.2).

Also, as an additional technical note, a challenge with any simulation study is how to select an appropriate reaction coordinate. In the case of chemical reactivity, geometric reaction coordinates are often used to describe reaction progress. While such reaction coordinates may easily be used for simple systems such as modelling reactions occurring in vacuum or in the condensed phase, clearly it is challenging to describe enzymatic chemistry, which can involve significant rearrangement of not just reacting atoms but also the surrounding enzyme as the reaction progresses, using a simple reaction coordinate. Here, we benefit from not using a standard geometric reaction coordinate; rather, the reaction coordinate used in our EVB calculations is the ‘energy gap’ (*x* = ∊_1_ − ∊_2_) between the different diabatic states (Warshel, 1991 ▸), which allows us to project the full multidimensional space of the enzymatic system onto a one-dimensional reaction coordinate. In doing so, it allows us to account for both the system reorganization and also the solute response to the solvent polarization, which is important when it comes to the screening of mutations, since a protein will respond to change in the local electrostatic environment. We note that it has been argued elsewhere that it would be impossible to correctly quantify the catalytic effect of different amino-acid substitutions without capturing these reorganization effects (see also Frushicheva *et al.*, 2014 ▸; Fuxreiter & Mones, 2014 ▸). To summarize, therefore, EVB is a very powerful approach for the computational screening of large numbers of enzyme variants because it is fast and efficient, allowing the extensive sampling necessary to obtain converging free-energy calculations, while capturing the reorganization energies (which would be simply too computationally expensive using *ab initio* quantum-mechanical approaches). In addition, as it is based on rigorously parameterized force fields, the EVB approach carries sufficient chemical information to describe chemical reactivity in a physically meaningful way.

**Figure 3:**
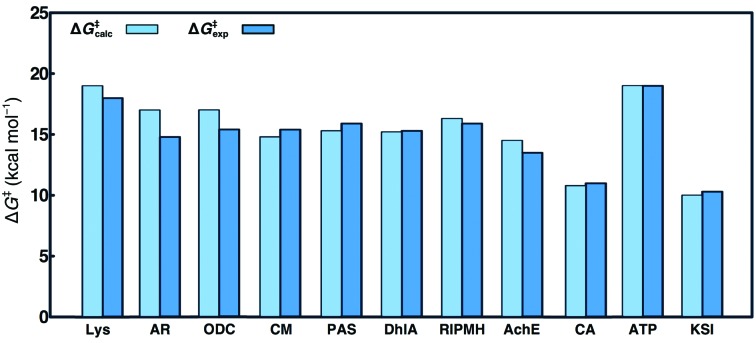
A comparison of calculated (using EVB, Δ*G* ^‡^_calc_) and experimentally observed (Δ*G* ^‡^_exp_) activation free energies for the reactions catalyzed by dihydrofolate reductase (DHFR), lysozyme (Lys), aldose reductase (AR), chorismate mutase (CM), trypsin (Try), a bacterial arylsulfatase (PAS), haloalkane dehalogenase (DhlA), triosephosphate isomerase (TIM), a bacterial phosphonate monoester hydrolase (RlPMH), acetyl­choline esterase (AchE), orotidine monophosphate decarboxylase (ODC), carbonic anhydrase (CA), F_1_-ATPase (ATP) and ketosteroid isomerase (KSI). This figure was prepared based on data presented in Warshel *et al.* (2006 ▸), Kamerlin *et al.* (2010 ▸), Luo *et al.* (2012 ▸) and Barrozo *et al.* (2015 ▸), and references cited therein.

Finally, and of particular importance to *CADEE*, the ability of well parameterized EVB force fields to reproduce the catalytic effect of broad ranges of wild-type and mutant enzymes has been well documented and thus the EVB approach provides a powerful tool for computational enzyme design (Warshel *et al.*, 2006 ▸; Frushicheva *et al.*, 2011 ▸; see Fig. 3 ▸ for concrete examples of this for different enzymes). As an aside, we would like to point out that the fact that EVB is a semi-empirical approach could be a concern for some users of high-level *ab initio* approaches. While we do agree that high-level approaches could in principle provide more precise results, the high cost of such methodologies at present prevents their application to massive computational screening, where it is not only necessary to compute the energetics of the reaction occurring in the active site of the wild-type enzyme and the energetics of experimentally observed amino-acid substitutions, but then also to perform the same calculations for thousands of *in silico* amino-acid substitutions. This renders the usage of high-level *ab initio* approaches computationally very, if not even prohibitively, expensive, at least for the time being, although this will hopefully change with the constant advances in both computer power and method­ologies.

Finally, previous attempts at enzyme design using the EVB approach have either coupled the EVB calculations to empirical screening approaches based, for example, on consideration of residue charge contributions to calculated activation barriers (Roca *et al.*, 2009 ▸; Frushicheva *et al.*, 2010 ▸; Labas *et al.*, 2013 ▸) or have screened comparably limited numbers of explicit amino-acid substitutions using EVB (Roca *et al.*, 2009 ▸; Frushicheva *et al.*, 2011 ▸). The key contribution of the present work is to provide a semi-automated framework within which to perform large ensembles of EVB calculations of many different enzyme variants simultaneously, thus greatly simplifying the computational design process.

### 2.2. The *CADEE* framework   

For simplicity, *CADEE* is a Python 2.7 application that interfaces with external programs such as *SCWRL*4 (Krivov *et al.*, 2009 ▸) as well as local analysis scripts in order to automatically generate EVB inputs for a large number of enzyme variants and to perform the associated EVB calculations with *Q* and subsequent analysis. The *CADEE* interface and associated analysis scripts are all available for download from Github at http://www.github.com/kamerlinlab/cadee. The actual molecular-dynamics equilibration and subsequent EVB free-energy calculations are performed using the *Q* simulation package (Marelius *et al.*, 1998 ▸) as described in §[Sec sec2.3]2.3. *Q* is free for academic users, and information on how to obtain a license is provided at http://www.icm.uu.se/cbbi/aqvist-lab/q. Any user holding a *Q* license through the main developers is welcome to compile *Q* or contact the corresponding author for the compiled executable (see http://www.icm.uu.se/cbbi/aqvist-lab/q for more information).

**Figure 4:**
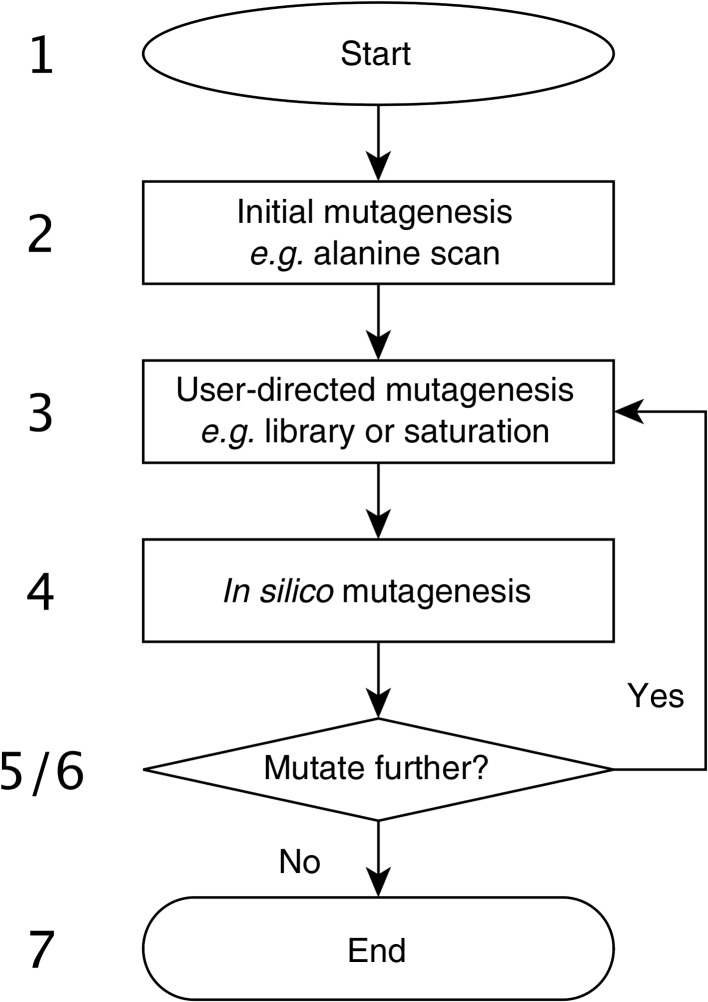
*CADEE* workflow. Basic input files are supplied to *CADEE* (1) and initial screening mutagenesis such as a computational alanine scan is performed (2). Automated analysis of the results is then performed and the user chooses which mutation hotspots should be mutated to which library (3). The next round of *in silico* evolution is then started (4) and automatically analyzed (5). Depending on the user input, (6) another round of evolution is performed (3–6) or the process is stopped (7) if the results are considered satisfactory.

As described in §[Sec sec2.1]2.1, efficient use of *CADEE* requires a well calibrated reference state, ideally benchmarked against the effect of a number of experimentally characterized amino-acid substitutions, following the standard EVB workflow (see, for example, Warshel *et al.*, 2006 ▸; Kamerlin & Warshel, 2011 ▸; Amrein *et al.*, 2015 ▸; Bauer *et al.*, 2016 ▸). This is the main limiting factor in the *CADEE* setup, as the quality of the *CADEE* runs will only be as good as the quality of the EVB force field used to perform the simulations, and therefore the rigorous parameterization of the EVB potentials involved is the most important step (and thus the greatest bottleneck) in the *CADEE* process. Once the user has parameterized an appropriate EVB force field using standard parameterization approaches compatible with the protein force field used, it is possible to use this as a baseline to produce an array of simulation inputs for simulating a user-defined range of enzyme variants. Structurally, therefore, *CADEE* consists of three major parts. The first of these is responsible for the generation of input files for all enzyme variants, as well as the relevant preparations for the simulations such as generating topology files, solvation of the system and any other input files necessary to prepare the simulation packages (simpacks). The second part of *CADEE* involves using *Q* (§[Sec sec2.3]2.3) and an mpi4py interface (Dalcin *et al.*, 2005 ▸) in order to organize and execute all available simpacks in parallel. The final part involves the analysis and presentation of the *CADEE* results to the user *via* a graphical analysis interface that allows rapid and straightforward selection of variants for subsequent rounds of *in silico* directed evolution (see Supplementary Fig. S1 for a screenshot of the analysis interface). We note also that while we have implemented *CADEE* to be mainly performed using command-line execution, we note that an exhaustive graphical user interface has recently been independently developed for *Q* (Isaksen *et al.*, 2015 ▸), which could aid the user further in simulation preparation and analysis. The basic workflow of *CADEE* is shown in Fig. 4 ▸.

We note as an aside that during the initial simulation setup stage, *CADEE* interfaces with *SCWRL*4 (Krivov *et al.*, 2009 ▸) to perform automated mutagenesis. We chose *SCWRL*4 because it is able to automatically resolve steric clashes upon substitution of an amino acid, because it is freely available for academic users and because it is very fast. However, as an alternative, amino-acid side chains can also be deleted (*e.g.* computational alanine scanning). The simulation input, including topology and other necessary files, is packed and subjected to our *Q* wrapper. In the next section, we will explain how the simulations are prepared and run on the available computational resources.

### 2.3. Interaction of *CADEE* with the *Q* simulation package   

Our main workhorse for performing the *CADEE* simulations is the *Q* simulation package, which was developed at Uppsala University by Åqvist and coworkers (Marelius *et al.*, 1998 ▸). In its current release version, v.5.0, *Q* is capable of performing standard molecular-dynamics (MD) simulations as well as free-energy perturbation (FEP), empirical valence-bond (EVB) and linear interaction energy (LIE) calculations. A key benefit of using *Q* for performing the EVB simulations is the availability of a wide range of force fields to choose from (see http://www.icm.uu.se/cbbi/aqvist-lab/q), as well as the implementation of Warshel’s local reaction field (LRF) approach (Lee & Warshel, 1992 ▸) to accurately represent electrostatic effects in enzymatic systems using truncations with stochastic boundary conditions, which greatly reduces simulation time.

**Figure 5:**
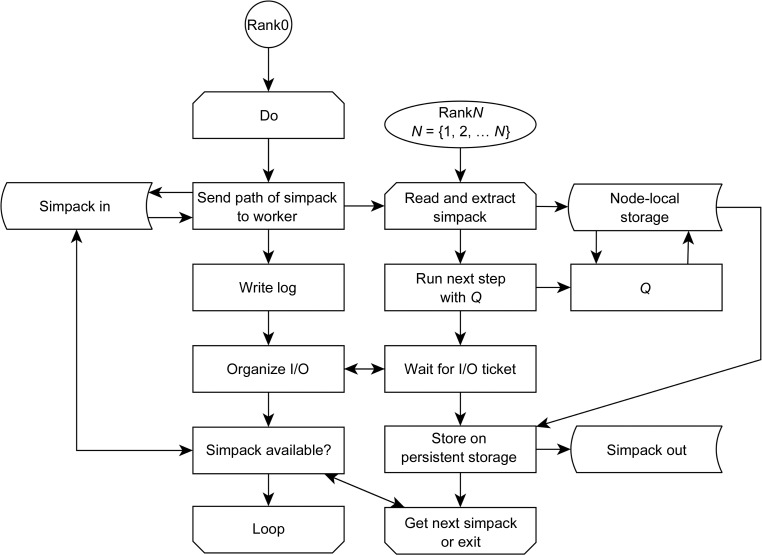
*CADEE* ensemble-simulation flowchart. When *CADEE* is initialized, it first locates all simpacks available in the initialization directory. It then distributes them on the available resources and runs each step of a simpack using *Q*. After completion of the simpack, the next simpack is executed. Individual simpacks are independent of each other and are executed in parallel, provided that multiple cores are available on the system/allocation.

The code base of *Q* is written in Fortran90. In order to increase portability to other approaches, rather than modifying *Q* directly, we created a Python framework that allows us to run ensemble simulations without overwhelming the file system with excessive input/output (I/O) requests (the code can be found at http://www.github.com/kamerlinlab/cadee). For this, we implemented an mpi4py-based wrapper (Dalcin *et al.*, 2005 ▸), which controls job I/O, schedules simpacks and then runs the simulations using *Q*. This wrapper is written in simple and straightforward Python. After an allocation becomes available, one simpack after another is processed. After a simpack is completed, the next simpack is loaded, unpacked and computed (see Fig. 5 ▸). When all simulations are finished, the analysis is performed and the user can initiate display of the results. The user in turn can then select new amino-acid substitutions to be tested on the selected protein (*e.g.* a site-saturated mutagenesis on an interesting residue), or they can choose to stop and save the results at this stage.

### 2.4. Model system: triosephosphate isomerase   

**Figure 6:**
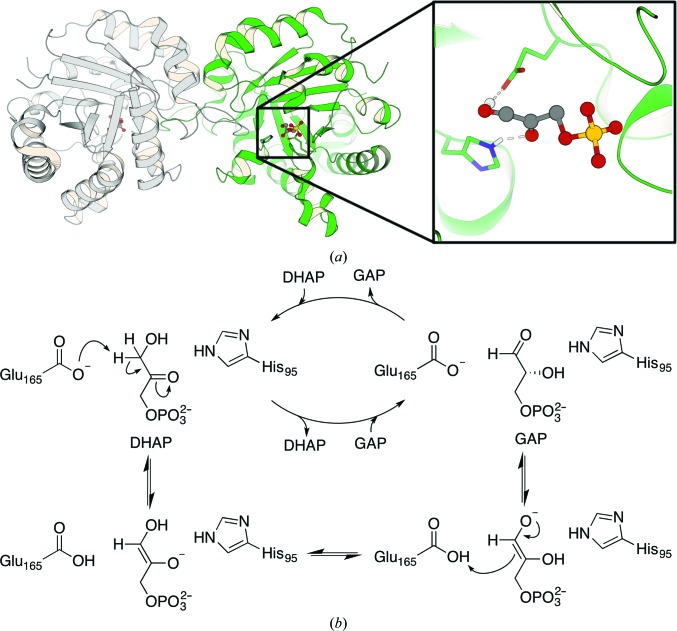
(*a*) Left, an overview of the structure of triosephosphate isomerase from *S. cerevisiae* (PDB entry 1ney; Jogl *et al.*, 2003 ▸) in complex with DHAP (displayed in ball-and-stick representation and coloured dark grey). Right, a close-up view of the active site, with highlighted key catalytic residues His95 and Glu165 and the substrate DHAP. Chain *A* is coloured green and chain *B* light grey. (*b*) The proposed mechanism for the isomerization catalysed by TIM. DHAP and GAP are acronyms for dihydroxyacetone phosphate and (*R*)-glyceraldehyde 3-phosphate, respectively. This mechanism is based on Wierenga *et al.* (2010 ▸) and Richard (2012 ▸).

We chose the enzyme triosephosphate isomerase (TIM) from *Saccharomyces cerevisiae* as our model system owing to the availability of a high-resolution crystal structure of a TIM–DHAP complex (1.2 Å resolution; PDB entry 1ney; Jogl *et al.*, 2003 ▸; Fig. 6 ▸ *a*) in the Protein Data Bank (Berman *et al.*, 2000 ▸). We note as an aside, however, that lower resolution crystal structures can also be successfully applied as templates for EVB simulations (see, among other examples, Trobro & Åqvist, 2006 ▸), but in such cases the simulation times should be increased to compensate for the uncertainty in the atomic coordinates. Similarly, greater conformational sampling is necessary for more flexible or thermally unstable systems.

TIM is a homodimer in most characterized enzymes (a dimer of dimers was found for enzymes from thermophiles), with the active sites at the dimer interface (Fig. 6 ▸ *a*). It catalyzes a simple reversible isomerization of di­hydroxyacetone phosphate (DHAP) and (*R*)-glyceraldehyde 3-phosphate (GAP) (Fig. 6 ▸ *b*). TIM is found in nearly every organism, as it is essential for glycolysis (Wierenga *et al.*, 2010 ▸). The overall chemical reaction, a 3,2-proton shift, proceeds *via* two enediolate intermediates (Wierenga *et al.*, 2010 ▸; Richard, 2012 ▸) and involves the catalytic residues Glu165 and His95 as acid/base catalysts (*S. cerevisiae* enzyme residue numbering, UniProt ID P00942; DHAP C-atom numbering as in Supplementary Fig. S2). In the (rate-limiting) first reaction step of the isomerization of DHAP to GAP, Glu165 deprotonates C3 of DHAP to form the 2-enediolate, which is then isomerized by a proton transfer from O3 to O2. Finally, C2 is protonated by Glu165 to form the aldose isomer GAP. For computational simplicity, in this work we have focused our computational effort only on the initial proton transfer from the C3 atom of DHAP to the Glu165 side chain, as the purpose of these calculations are purely pedagogical in order to illustrate how *CADEE* works.

The structure used as the starting point for our simulations (PDB entry 1ney; Jogl *et al.*, 2003 ▸) contains three amino-acid substitutions, which were initially introduced to enable fluorescence probing experiments (Sampson & Knowles, 1992 ▸). However, these amino-acid substitutions were demonstrated experimentally to neither change the kinetic properties of the enzyme nor alter its structural fold (Rozovsky *et al.*, 2001 ▸), and therefore this structure was used as the starting point for all simulations in this work. For the purpose of our simulations, we retained the W90Y and W157F substitutions present in the crystal structure, but changed residue 168 back from 5′-fluorotryptophan to the canonical tryptophan; this double mutant will be referred to as our ‘wild-type’ (WT) system in the following discussion, as it forms the baseline for all subsequent *CADEE* simulations. All water molecules outside the simulation sphere (20 Å around C1 in the bound DHAP in chain *A*, see below) were removed and the protonation states of the histidine side chains were evaluated using the *MolProbity* server (Chen *et al.*, 2010 ▸). The protonation states of all other ionizable residues within 17 Å of the sphere centre were determined by their p*K*_a_ values in solution and by visual inspection (see Supplementary Table S1). All residues outside the 17 Å sphere were kept uncharged, as is standard procedure for such simulations (Labas *et al.*, 2013 ▸; Amrein *et al.*, 2015 ▸; Lameira *et al.*, 2015 ▸; Isaksen *et al.*, 2016 ▸).

Amino-acid substitutions were introduced into the WT TIM structure with *CADEE*, either with the internal alanine-scanning method or *via* the use of *SCWRL*4 (Krivov *et al.*, 2009 ▸) using the standard settings of the software and preventing the atoms of the EVB region from being altered by *SCWRL*4. All other conformational changes suggested by *SCWRL*4 were applied and both the WT and the modified enzymes were solvated as described above. The modified enzymes were then equilibrated as described for the WT enzyme, as described in §§[Sec sec2.4.1]2.4.1 and [Sec sec2.4.2]2.4.2.

#### 2.4.1. Molecular-dynamics simulations   

All simulations were performed using the *Q* simulation package (v. 5.06) with the OPLS-AA force field (Jorgensen *et al.*, 1983 ▸). OPLS-AA compatible parameters for the DHAP ligand and the enediolate intermediate were obtained using *MacroModel* 9.1, force field version 2011 (release 2013-1: Schrödinger, 2013 ▸), and the corresponding partial charges were calculated in the gas phase at the HF/6-31G* level of theory, using the standard RESP procedure and *Gaussian*09 rev. D.01 (Cieplak *et al.*, 1995 ▸; Frisch *et al.*, 2009 ▸).

In order to prepare TIM for our EVB simulations, the simulation system was first solvated in a spherical water droplet of TIP3P water molecules (Jorgensen *et al.*, 1983 ▸) with a radius of 20 Å, centred on the C1 atom of the DHAP substrate (see Supplementary Fig. S2 for C-atom numbering in DHAP). The droplet was described by spherical boundary conditions, using the surface-constrained all-atom solvent SCAAS model (King & Warshel, 1989 ▸) as implemented in *Q* (Marelius *et al.*, 1998 ▸). For computational simplicity, as with our previous work (Amrein *et al.*, 2015 ▸; Barrozo *et al.*, 2015 ▸; Ben-David *et al.*, 2015 ▸), we used a multi-layer model in which all atoms within 17 Å of the simulation centre were fully mobile, all atoms between 17 and 20 Å of the simulation centre were restrained using a 10 kcal mol^−1^ Å^−2^ harmonic restraint and atoms outside 20 Å were restrained by a harmonic force constant of 200 kcal mol^−1^ Å^−2^. The *SHAKE* algorithm (Ryckaert *et al.*, 1977 ▸) was used to constrain H atoms in the solvent. A 10 Å cutoff was used for calculating nonbonded interactions between all atoms except for those in the EVB region (the catalytic glutamate residue and the substrate), for which all interactions were explicitly calculated up to 99 Å (*i.e.* essentially no cutoff was applied). All long-range electrostatics beyond this cutoff were treated using the local reaction field (LRF) method (Lee & Warshel, 1992 ▸).

Once the system setup was complete, the systems were heated gradually from 0.01 to 300 K over the course of 90 ps of simulation time, starting with a 200 kcal mol^−1^ Å^−2^ harmonic restraint on all protein atoms and 20 kcal mol^−1^ Å^−2^ on all water atoms in the simulation sphere, and then gradually decreasing this from 0 kcal mol^−1^ Å^−2^ as the temperature was increased. The temperature was regulated using the Berendsen thermostat (Berendsen *et al.*, 1984 ▸; 100 fs bath coupling). A 1 fs time step was used and the reaction coordinate was set to λ = 0.5 for all simulations to start the subsequent EVB calculations of the reaction step close to the transition state. For each of four individual replicas we performed an 8 ns molecular-dynamics simulation, taking a snapshot every 1 ns, which was used as a starting point for an EVB simulation (see §[Sec sec2.4.2]2.4.2). Note that in order to calibrate our EVB parameters to model the TIM-catalyzed proton-transfer reaction, we also performed a corresponding simulation of the uncatalyzed proton transfer between DHAP and butanoate in aqueous solution, as described in §[Sec sec2.4.2]2.4.2.

#### 2.4.2. Empirical valence-bond calculations   

As mentioned in §[Sec sec1]1, only the first step of the TIM mechanism from DHAP was modelled in this study and was described in terms of two valence-bond states as depicted in Supplementary Fig. S2. All EVB calculations were performed utilizing the standard EVB free-energy perturbation/umbrella sampling (EVB-FEP/US) procedure as described in §[Sec sec2.1]2.1 and in Hwang *et al.* (1988 ▸) and Warshel (1991 ▸). As shown in Supplementary Fig. S2, the EVB (reacting) region consisted of the side chain of Glu165 (TIM) or the carboxylate group of butanoate (aqueous solution) and the DHAP molecule. The entire system was described using the same force field, however, and the only difference between atoms defined as EVB and non-EVB atoms in our simulations were the application of different cutoffs, as mentioned above, as well as the fact that Morse rather than harmonic potentials were used to describe the bonds that were broken or formed during the reaction. All EVB parameters used in the present work can be found in the Supporting Information for this article, and the overall methodology has been described in detail in our previous studies (Amrein *et al.*, 2015 ▸; Barrozo *et al.*, 2015 ▸). The background reaction in aqueous solution was parameterized to reproduce an activation free energy, Δ*G* ^‡^, of 25.2 kcal mol^−1^, and a reaction free energy, Δ*G*_0_, of 17.8 kcal mol^−1^, following Åqvist & Fothergill (1996 ▸), and the EVB simulations were performed using the same simulation settings as the initial equilibration runs.

In order to optimize the simulation time, we were interested in examining whether we could reproduce experimental values with rather short EVB runs, as it has recently been suggested that shorter simulations can have better prediction capability for the effects of amino-acid substitutions than longer ones (Wijma *et al.*, 2014 ▸). We therefore took snapshots every 1 ns of the 8 ns long MD simulation, and ran an EVB simulation of 520 ps in length, distributed over 26 EVB-FEP/US windows of 20 ps each (λ = 0, 0.05, 0.075, 0.1, 0.125, 0.15, 0.2, 0.25, 0,30, 0.35, 0.40, 0.425, 0.45, 0.55, 0.575, 0.6, 0.65, 0.70, 0.75, 0.80, 0.85, 0.875, 0.90, 0.925, 0.95, 1); additionally, for the mapping of the data of each snapshot, we used the data of the preceding 1 ns MD simulation (where λ = 0.5) to achieve an increased sampling close to the transition state. The EVB snapshots of the first 2 ns of MD simulation were discarded as they are taken during the initial equilibration of the system. Therefore, from within 8 ns of MD simulation six EVB snapshots per replica (24 in total, from four replicas) were used to calculate the mean values presented in §[Sec sec3]3. Each simulation was repeated four times with four different sets of initial velocities (random seeds), leading to a total of 12.48 ns of EVB simulation time per system.

Finally, all simulations of the uncatalyzed reaction in aqueous solution were performed in exactly the same way as for the TIM-catalyzed reaction, although a slightly different setup was used for simulating this reaction. That is, in this case, after stepwise heating the system up over the course of 260 ps, we performed 1 ns of equilibration at the transition state and then ran ten individual trajectories from the transition state, using 200 ps of simulation time per frame (leading to 10.2 ns of simulation time per trajectory and 102 ns of simulation time in total). The individual trajectories were generated by taking the end point of the initial equilibration run and performing an additional 1 ns of equilibration with a new random seed before starting the EVB simulation. These longer simulations were necessary as more sampling is required for the uncatalyzed reaction, where the reacting fragments can explore a larger conformational space, compared with the enzymatic reaction, where the fragments are restricted to the active-site cavity. As with the enzymatic reaction, a weak harmonic restraint was applied to all reacting atoms (in this case 1 kcal mol^−1^ Å^−2^) to prevent the reacting fragments from drifting too far from the reaction centre.

## 3. Results and discussion   

**Figure 7:**
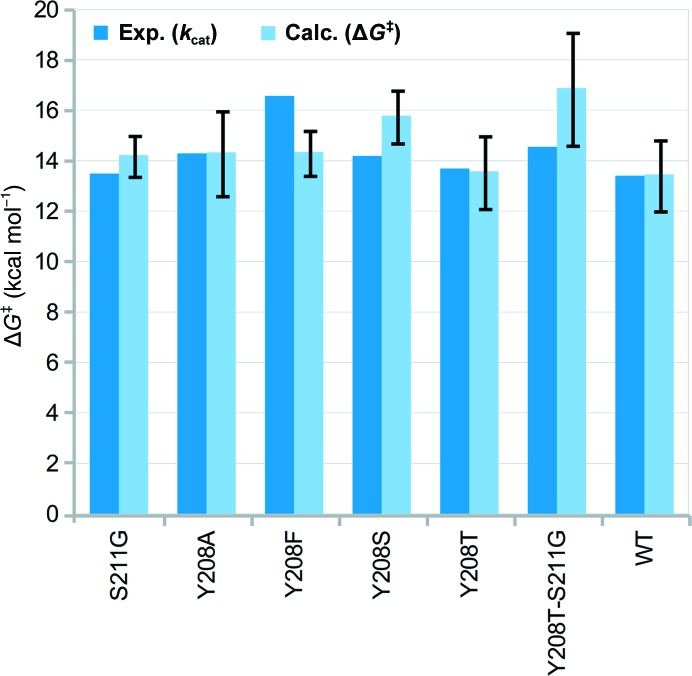
Experimental (*k*_cat_) and calculated activation free energies (Δ*G* ^‡^) for the deprotonation of DHAP by diverse *S. cerevisiae* TIM variants. Effects of amino-acid substitutions with experimental data from TIM enzymes of different organisms (chicken and *Trypanosoma brucei brucei*) were also calculated for the yeast enzyme but, as expected, gave less agreement (see Supplementary Fig. S3), illustrating that mutational effects cannot be easily transferred between enzymes with only around 50% sequence identity. Note also that substitutions in the chicken enzyme shown in Supplementary Fig. S3 involve His95, which is catalytically relevant in the subsequent reaction step. The experimental *k*_cat_ values were obtained at 25°C (Zhai *et al.*, 2015 ▸) and were used to estimate the Δ*G* ^‡^ of the rate-limiting step. The corresponding data can be found in Supplementary Table S2.

In this section, we will present a pedagogical example of the application of *CADEE* to triosephosphate isomerase (TIM). As mentioned before, clearly the initial parameterization is the most important part of any *CADEE* run and, as shown in Fig. 3 ▸, when well parameterized the EVB has an excellent track record of reliably reproducing catalytic effects in a broad range of biological systems (including enzymes with far poorer catalytic proficiency than TIM). In the present work, as we are only using proton transfer in TIM as a pedagogical example of the usage of *CADEE*, our aim was not to obtain perfect EVB potentials to describe this reaction, but rather to have a reasonable model to use with which to illustrate the *CADEE* workflow. For *CADEE* simulations to be physically meaningful, however, the starting point of any *CADEE* run should be rigorous validation of the EVB parameter set, which is best performed by benchmarking the parameter set against experimentally characterized mutations. Historically, TIM has been a very well studied system, with extensive biochemical data available in the literature. A comparison between our calculated and experimental results (using 24 short EVB simulations of 520 ps in length each, generated from four independent replicas, as described in §[Sec sec2.4.2]2.4.2) is shown in Fig. 7 ▸ and Supplementary Fig. S3, and the corresponding raw data are shown in Supplementary Table S2.

From this data, it can be seen that in most cases we can reproduce the trends in calculated activation free energies reasonably well and obtain calculated values within a maximum of 2 kcal mol^−1^ of the corresponding experimental activation free energies. Note that in these examples no catalytically crucial residues or their direct neighbours have been targeted, as one would expect such amino-acid substitutions to pose a particular challenge for predictions not only for *CADEE* but for all other methods as well (see Supplementary Fig. S3 for further discussion). Taking this into account, we find the predictions reasonable (when also considering current computational capabilities; Lind & Himo, 2013 ▸; Kaiyawet *et al.*, 2015 ▸; Lameira *et al.*, 2015 ▸; Świderek, Tuñón, Martí *et al.*, 2015 ▸) and note that of course the EVB potentials could have been further refined to give better agreement with experiment (as shown for other systems in Fig. 3 ▸), but the data shown in Fig. 7 ▸ are adequate for the purposes of illustrating the *CADEE* workflow.

**Figure 8:**
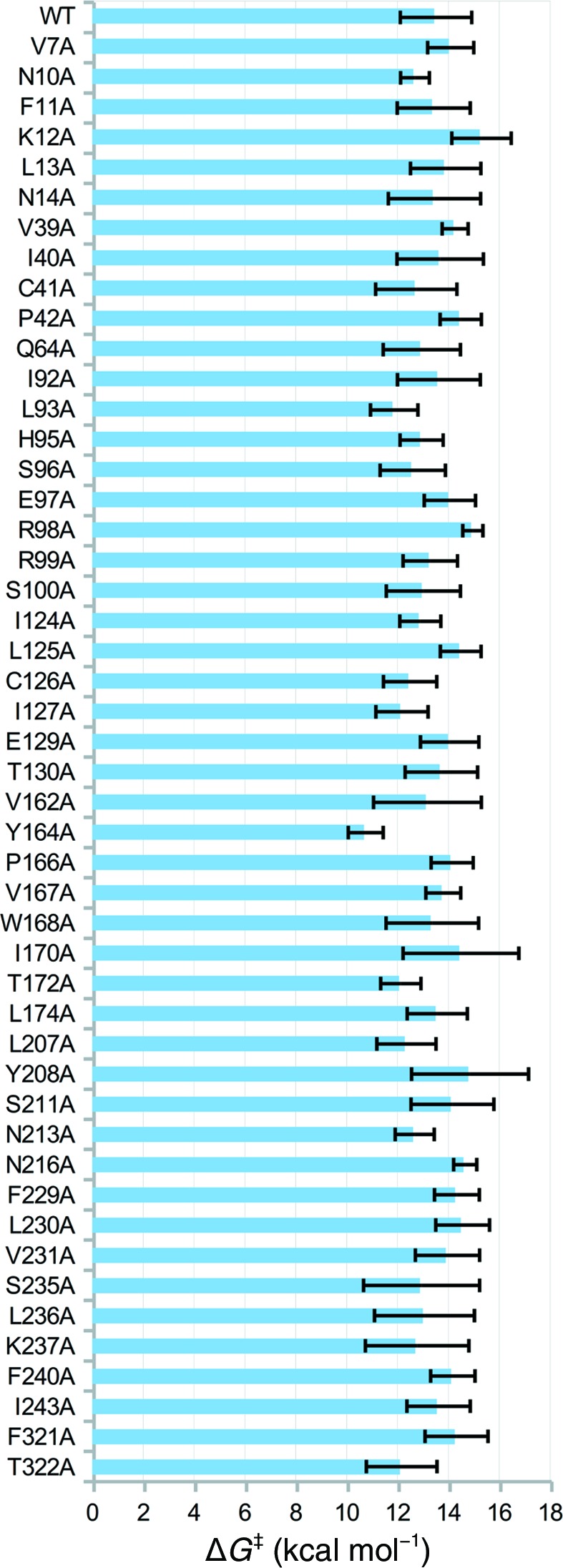
Results of the initial alanine scan. Activation energies (Δ*G* ^‡^) are given as median values, with error bars displaying the standard deviation over 24 individual EVB simulations. The corresponding raw data are provided in Supplementary Table S3.

**Figure 9:**
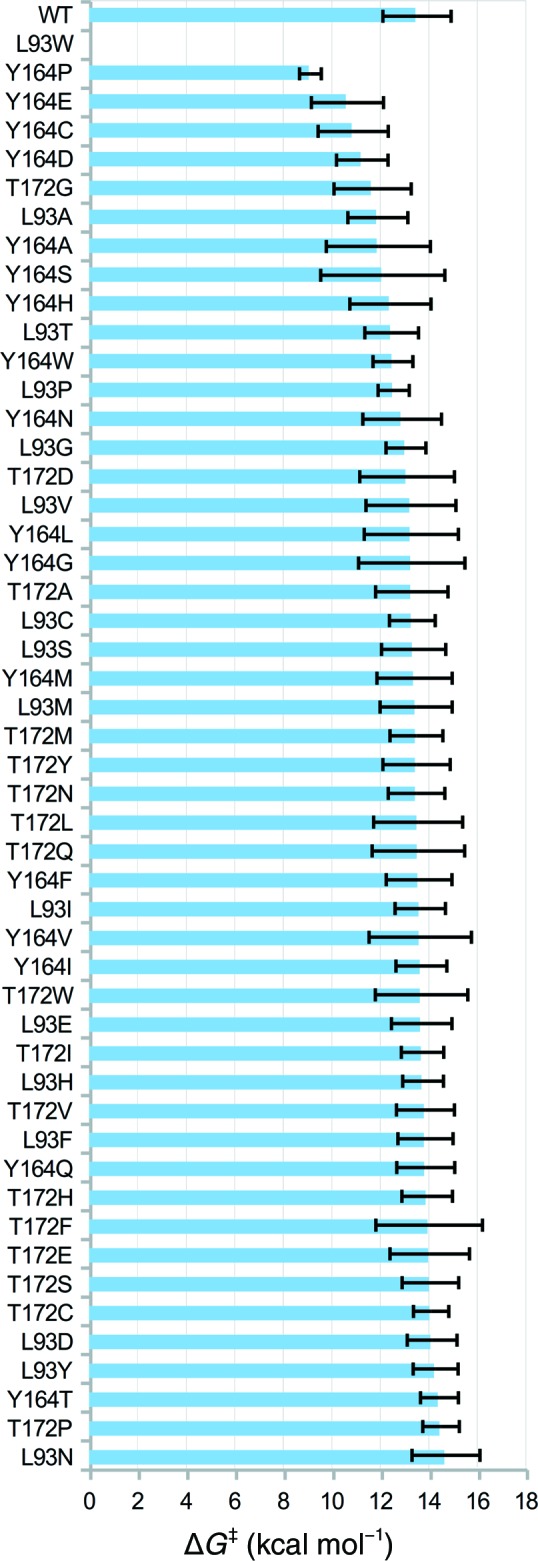
Results of the separate single-site saturation mutagenesis experiments at positions 93, 164 and 172. Activation energies (Δ*G* ^‡^) are given as mean values, with error bars showing the standard deviation over 24 individual EVB simulations. The corresponding raw data are provided in Supplementary Table S4.

Having verified that our EVB potential can reasonably reproduce known substitutions, we started by performing an alanine scan of the 48 non-catalytically crucial positions around the active site (see Supplementary Table S3). The results of this scan are shown in Fig. 8 ▸ and, based on this data, we selected the following three positions as ‘hotspots’ for further separate site-saturation mutagenesis: 93, 164 and 172 (Fig. 9 ▸). These particular positions were chosen because the introduction of an alanine there gave the lowest activation energy compared with the other positions; however, as can be seen from this figure, multiple residues are potential candidates for further mutagenesis, and therefore we recommend combining the computational alanine scanning performed using EVB with bioinformatics approaches based on protein sequence identity to select the best mutagenesis ‘hotspots’. Additionally, although at a first glance predictions with a standard deviation of around 2 kcal mol^−1^ (corresponding to around two orders of magnitude in *k*_cat_) might look uncertain, laboratory high-throughput screening or selection methods (Packer & Liu, 2015 ▸) almost never allow direct conclusions on the kinetic properties of tested variants and, additionally, the standard deviations there are comparably large as well. Therefore, *CADEE* is likely to provide valuable guidance for predicting mutations for laboratory testing (for a more thorough cost–benefit analysis, see below).

From this figure (and also from Figs. 8 ▸ and 10 ▸), it can be seen that there is a quite broad spectrum of predictions from *CADEE*, which in some cases also suggest fairly radical reductions of the activation free energy relative to the wild-type enzyme. A weakness of *CADEE* is the risk of obtaining the ‘right answer’ for the wrong reason. That is, specifically, it is possible to have disrupted the active site and/or thus also destabilized the ground state when introducing an *in silico* amino-acid substitution, in this way calculating an artificially low activation free energy that is not physically meaningful (this is not a unique problem to EVB simulations, but is a problem for all current approaches that aim to model the effect of amino-acid substitutions on reaction chemistry through *in silico* mutagenesis). Therefore, if it appears to be ‘too good to be true’ it most likely is, and predictions of very radical reductions in activation free energy should probably be discarded or at least very carefully examined for simulation artefacts (such as major active-site perturbations) before proceeding to the next round of mutagenesis. Also, some changes might impact a different step of the reaction mechanism, which lies beyond the scope of the present study, but which, given a proper parameterization and sufficient computational resources, can also be examined with *CADEE*.

**Figure 10:**
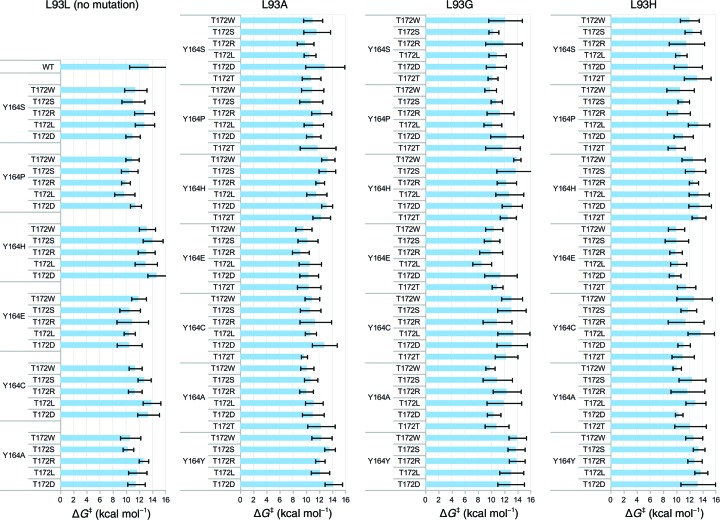
Results of the combinatorial saturation mutagenesis at positions 93, 164 and 172 grouped into columns based on the substitution at position 93 and further arranged into groups by substitutions at position 164. Activation energies (Δ*G* ^‡^) are given as the mean, with error bars showing the standard deviation over individual 24 EVB simulations. The corresponding raw data are provided in Supplementary Tables S5–S8.

Clearly, as in any laboratory directed-evolution experiment, this procedure can be repeated as many times as necessary, and further rounds of evolution are clearly going to be required in real-life scenarios when working with enzymes with lower catalytic proficiencies. This can therefore be continued as long as the user desires to further refine the results, including intermediate iterations of experimental validation, which can be brought into the cycle at any point. For illustrative purposes, therefore, we performed a final round of *in silico* mutagenesis, in which we tested modifying three positions simultaneously. Specifically, and following the data shown in Fig. 9 ▸, we again performed substitutions at positions 93, 164 and 172, this time modifying Leu93 to Ala, Gly and His, Tyr174 to Ser, Cys, Ala, His, Glu and Pro, and Thr172 to Leu, Trp, Asp, Arg and Ser simultaneously. These were chosen on the basis of their individual effect on the calculated activation barriers as found in the separate site-saturation mutagenesis experiments. Each of these substitutions individually lowered the predicted activation energy, and we wanted to rule out synergistic effects of these replacements. The resulting data, which are shown in Fig. 10 ▸, imply that mutating the positions 93, 164 and 172 simultaneously has a cooperative effect, as double and triple mutants were predicted to have lower activation barriers than all other variants of the separate site-saturation round. At this point in a real evolution study, and after visual inspection of the trajectories, one could experimentally evaluate selected predicted mutants and decide based on the outcome of experimental testing on how to continue in the next iteration.

We demonstrate, therefore, that when sufficient computational resources are available it is possible to perform large-scale combinatorial mutagenesis and also longer EVB simulations to obtain better sampling. We strongly recommend that when selecting final substitutions at every round, these are subjected to longer EVB runs to make sure that the results are not an artefact caused by the limited simulation time, thus also allowing the selected side-chain rotamers in the modified proteins more simulation time to properly equilibrate during the additional simulation time in order to reduce the risk that they represent catalytically inactive conformations. In the present case the relatively short runs could reproduce the experimental results reasonably well. However, we are looking at only a simple proton-transfer reaction; clearly the more complex the reaction the longer the runs necessary.

Following from this, *CADEE* has been deliberately constructed in such a way as to be scalable to the computational needs available. All equilibration and EVB runs shown in Figs. 7 ▸, 8 ▸, 9 ▸ and 10 ▸ were performed using a total of 225 000 core hours on the HPC2N Abisko cluster in Umeå (https://www.hpc2n.umu.se/resources/abisko), using nodes consisting of four AMD Opteron 6238 12-core 2.6 GHz processors per node. To provide a better estimate of the computational resources that a comprehensive *CADEE* run would need in a ‘real-world example’, we assume a system of 200 residues, in which all residues are to be substituted with alanine. We assume that after this initial scan ten positions will be picked and all 20 natural amino acids will be tested individually. Finally, for up to five of these positions, the user selects three residues that then will be scanned combinatorially. This setup would cost 520 000 core hours on a computer similar to the HPC2N Abisko cluster (AMD Opteron 6238 12-core 2.6 GHz processors). Compared with what it would cost to create the libraries in a laboratory and to screen them to achieve a full coverage of all these variants, *CADEE* is relatively cheap. In addition, to save computational time, we are only subjecting the most likely rate-limiting step of the reaction pathway shown in Fig. 6 ▸(*b*) to *CADEE* simulations. This can be identified either on the basis of available experimental studies or by using EVB or other computational tools to perform an initial screen of the full reaction pathway. We recommend, however, that the user performs EVB simulations of the full reaction pathway for the final variants in order to ensure that the mutations have not changed the rate-limiting step, making one step energetically favourable while negatively impacting another. Finally, as successfully shown for laboratory directed evolution (Verma *et al.*, 2012 ▸), *CADEE* can of course also be combined with computational tools for creating smarter libraries, to help predicting mutational hotspots or with machine-learning techniques to suggest additional variants from the results obtained thus far to further reduce the cost by simply screening libraries of reduced size. From this, it can be seen that *CADEE* is an excellent simple tool to perform (mostly) automated *in silico* directed evolution as a screening tool to aid laboratory design studies.

## 4. Summary and outlook   

Recent years have seen an explosion of interest in computational enzyme design, using both empirical screening and machine-learning approaches to predict the effect of individual amino-acid substitutions on the function and stability of an enzyme, as well as the development of new approaches for *de novo* enzyme design and *in silico* evolution (Verma *et al.*, 2012 ▸; Kiss *et al.*, 2013 ▸; Kries *et al.*, 2013 ▸; Damborský & Brezovský, 2014 ▸; Frushicheva *et al.*, 2014 ▸; Świderek, Tuñón, Moliner *et al.*, 2015 ▸). It has been shown elsewhere that the empirical valence-bond approach is a powerful aid in enzyme-design efforts, owing to the ability of a well parameterized EVB potential to predict the effect of different amino-acid substitutions on the catalytic activity of an enzyme (Warshel *et al.*, 2006 ▸; Frushicheva *et al.*, 2011 ▸). However, at present the process of setting up and analysing such simulations can be onerous, in particular when the intention is to screen for the effect of hundreds or thousands of amino-acid substitutions, which is necessary in a large-scale enzyme-design study.

In the present work, we introduce a new computational tool, *CADEE*, which allows user-controlled *in silico* directed evolution of enzymes. We apply *CADEE* to a very simple model system, triosephosphate isomerase, as a pedagogical example of how *CADEE* can be applied in a computational enzyme-design study. We demonstrate that for this simple system we are easily able to screen the effect of 128 amino-acid substitutions in 9.5 d using 512 processor cores of AMD Opteron 6238 clocked at 2.6 GHz (or 120 000 core hours). If Intel architecture is used instead, for example Xeon E5-2660 clocked at 2.2 GHz, the calculation time would decrease to 6.5 d using the same number of cores (80 000 core hours). We selected these resources for benchmarking purposes, as we believe this is a level of computer power that should be available to most research teams. In addition, *CADEE* has been constructed so that it can be up-scaled or down-scaled according to user needs and resources. Finally, while in its current implementation *CADEE* has been designed to perform empirical valence-bond calculations, clearly the principle of *CADEE* can be applied to any computational approach for modelling enzyme activity that provides sufficient accuracy with acceptable computational cost to the user. We believe, therefore, that *CADEE* will provide a valuable resource to users interested in performing *in silico* directed evolution as well as a useful aid to laboratory evolution studies.

## 5. Related literature   

The following reference is cited in the Supporting Information for this article: Zhai *et al.* (2013 ▸).

## Supplementary Material

Supporting Information.. DOI: 10.1107/S2052252516018017/be5274sup1.pdf
