# Supplementary material for: CADEE: Computer-Aided Directed Evolution of Enzymes
Source: IUCrJ. 2017 Jan 1;4(Pt 1):50–64. doi: 10.1107/S2052252516018017 (PMC5331465; doi:10.1107/S2052252516018017)
Supplement: Supplementary file 1 [file m-04-00050-sup1.pdf]

IUCrJ

Volume 4 (2017)

Supporting information for article:

CADEE: Computer-Aided Directed Evolution of Enzymes

Beat Anton Amrein, Fabian Steffen-Munsberg, Ireneusz Szeler, Miha Purg, Yashraj Kulkarni and Shina Caroline Lynn Kamerlin

S1. Supplementary figures

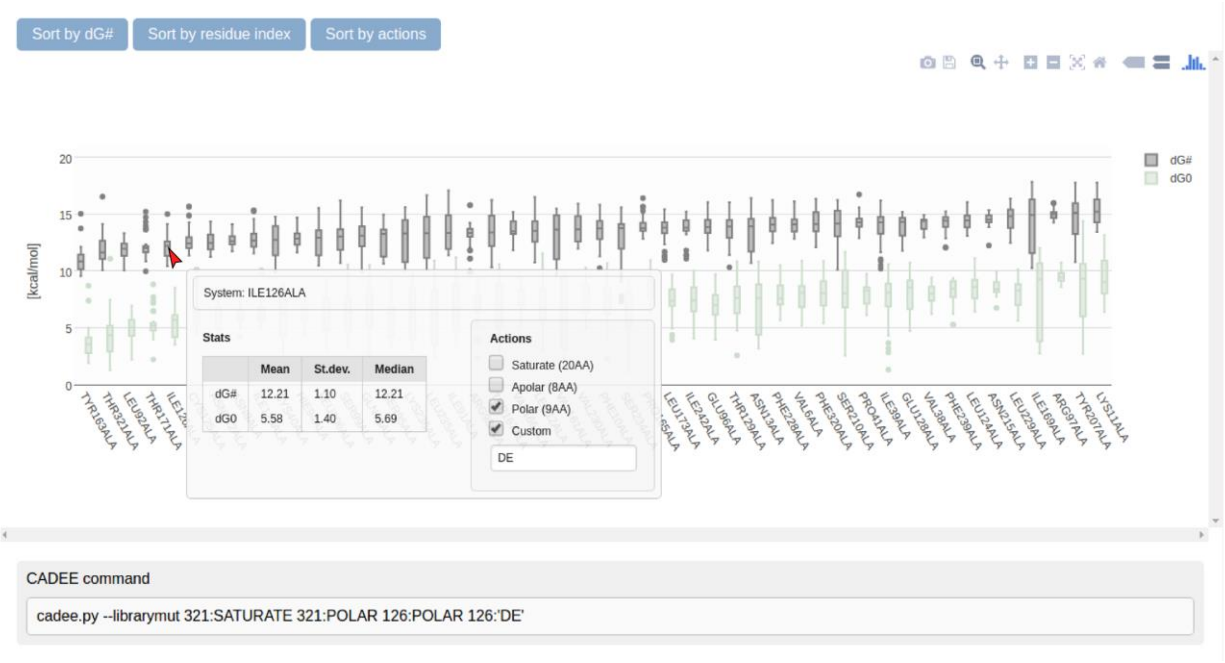

Figure S1 A screen shot of the CADEE graphical user interface, showing the alanine scan data presented in Fig. 8 and Table S3. The data is plotted as boxplots, with the selection tool open for illustrative purposes. The selection tool displays the option menu for the residue at position 126.

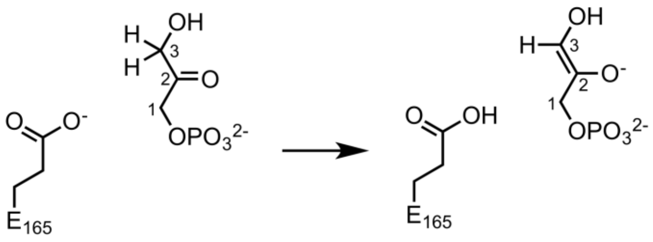

I II

Figure S2 Structures of the different VB states used in this work, and the C atom numbering.

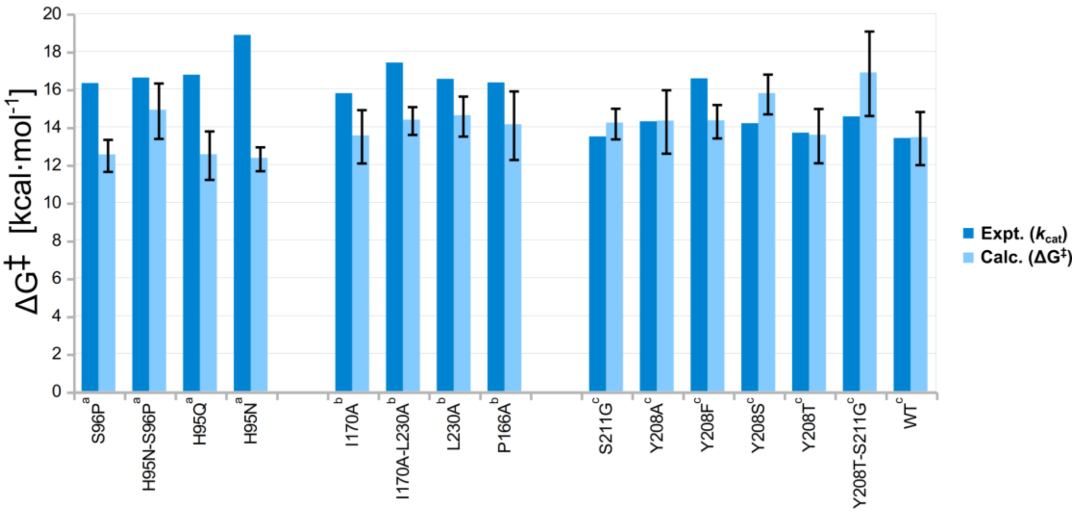

Figure S3 Experimental (k_{cat}) and calculated activation free energies ($\Delta G^{\ddagger}$) for the deprotonation of DHAP by diverse TIM variants (this is an extended version of Fig. 7 of the main text, now including also comparison to other organisms). The experimental data is arranged with respect to the organism and the residue numbering was adapted to the corresponding position in the *Saccharomyces cerevisiae* enzyme. The calculated values have all been obtained with the corresponding amino acid substitutions in the yeast enzyme, and therefore the larger discrepancy to the experimental values in these cases is therefore not surprising. It clearly illustrates that mutational effects cannot directly be transferred among enzymes from different organisms (the enzymes from chicken and *Trypanosoma brucei brucei* share 52% and 50% sequence identity with the yeast enzyme, respectively). Additionally, substitutions in H95, which is a catalytically critical residue in the second step (Richard, 2012), most likely lead to a drastic increase in the activation free energy in the second step as this is the step where this residue is most catalytically relevant (see mechanism in Fig. 6(b) of the main text) and thereby making it rate-limiting, which cannot be taken into account when only modelling the first step. Furthermore, the high discrepancy for the S96P mutation can be rationalized by a structural perturbation of the active site (data not shown). Note that substitutions in the chicken enzyme involve H95 which is catalytically relevant in the subsequent reaction step (Richard, 2012). [a)] Experimental k_{cat} from *Gallus gallus domesticus*, obtained at 30 ºC (Blacklow & Knowles, 1990). [b)] Experimental k_{cat} from *Trypanosoma brucei brucei*, obtained at 25 ºC (Malabanan *et al.*, 2011, Zhai *et al.*, 2013, Richard *et al.*, 2016). [c)] Experimental k_{cat} from *Saccharomyces cerevisiae*, obtained at 25 ºC (Zhai *et al.*, 2015). The corresponding data can be found in Table S2.

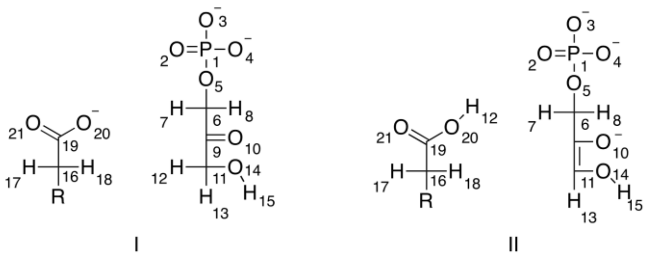

Figure S4 Atom numbering in the different VB states (corresponding to the numbering used in the EVB parameter tables provided in Section S2).

Table S1 List of residues that were ionized in the simulations, as well as the protonation patterns of histidine residues. All other residues were outside the simulation sphere and were thus kept in their unionized forms (see also Section 2.4 of the main text).

Residue	Residue number
Asp	-
Glu	97, 129, 165, 239
Lys	12, 237
Arg	98, 99
His-δ	103, 185, 350, 432
His-ε	95, 342

Table S2 Summary of the experimental (Expt.) and corresponding calculated (Calc.) activation free energies, $\Delta G^{\ddagger}$, for various TIM variants.[a)]

Mutant	Expt.	Calc.	Median	Standard deviation	Standard error of mean
S96P	16.31	12.53	12.69	0.85	0.17
H95N/S96P	16.60	14.89	14.48	1.46	0.30
H95Q	16.75	12.54	12.45	1.28	0.26
H95N	18.86	12.35	12.34	0.63	0.13
I170A	15.77	13.54	13.52	1.42	0.29
I170A/L230A	17.40	14.37	14.40	0.74	0.15
L230A	16.54	14.61	14.54	1.05	0.21
P166A	16.34	14.13	14.37	1.81	0.37
S211G	13.49	14.22	14.25	0.81	0.17
Y208A	14.29	14.32	14.15	1.68	0.34
Y208F	16.56	14.34	14.28	0.89	0.18
Y208S	14.18	15.78	15.85	1.05	0.21
Y208T	13.68	13.57	13.78	1.44	0.29
Y208T/S211G	14.55	16.87	17.54	2.24	0.46
WT	13.40	13.45	13.72	1.40	0.12

[a)] This data is also presented in visual form in Figs. 7 and S3. Experimental data based on (Blacklow & Knowles, 1990, Malabanan *et al.*, 2011, Zhai *et al.*, 2013, Zhai *et al.*, 2015, Richard *et al.*, 2016). All energies are shown in kcal·mol^{-1}. All calculated values presented here are averages and standard deviations / standard error of the mean over 24 individual trajectories.

Table S3 Calculated activation free energies from the initial alanine scan. [a)]

Mutant	Average	Median	Standard deviation	Standard error of mean	Mutant	Average	Median	Standard deviation	Standard error of mean
T322A	12.10	11.64	1.39	0.29	I127A	12.11	12.22	1.03	0.21
F321A	14.26	14.10	1.24	0.25	C126A	12.43	12.12	1.04	0.21
I243A	13.55	13.77	1.24	0.25	L125A	14.43	14.22	0.80	0.17
F240A	14.11	14.27	0.87	0.18	I124A	12.84	12.68	0.81	0.16
K237A	12.71	12.72	2.03	0.41	S100A	12.96	13.01	1.46	0.30
L236A	13.00	13.34	1.97	0.40	R99A	13.23	13.37	1.07	0.22
S235A	12.89	13.72	2.28	0.47	R98A	14.90	14.85	0.40	0.08
V231A	13.90	13.68	1.26	0.30	E97A	14.00	13.90	1.01	0.21
L230A	14.50	14.79	1.06	0.22	S96A	12.54	13.28	1.28	0.27
F229A	14.27	14.21	0.88	0.18	H95A	12.89	12.81	0.85	0.18
N216A	14.60	14.49	0.46	0.09	L93A	11.81	12.02	0.93	0.19
N213A	12.62	12.72	0.76	0.16	I92A	13.57	13.48	1.63	0.33
S211A	14.10	14.60	1.63	0.33	Q64A	12.89	12.83	1.52	0.31
Y208A	14.79	15.30	2.30	0.48	P42A	14.42	14.39	0.81	0.16
L207A	12.29	12.50	1.16	0.24	C41A	12.68	13.05	1.60	0.33
L174A	13.50	13.84	1.19	0.24	I40A	13.62	14.11	1.70	0.35
T172A	12.06	11.93	0.79	0.16	V39A	14.20	14.38	0.51	0.12
I170A	14.43	15.24	2.28	0.46	N14A	13.39	13.90	1.81	0.37
W168A	13.31	13.48	1.82	0.37	L13A	13.83	13.77	1.38	0.28
V167A	13.74	13.45	0.69	0.16	K12A	15.24	15.06	1.17	0.24
P166A	14.09	13.85	0.83	0.17	F11A	13.36	13.71	1.43	0.29
Y164A	10.69	10.86	0.69	0.14	N10A	12.63	12.58	0.56	0.12
V162A	13.12	13.76	2.12	0.50	V7A	14.03	13.86	0.91	0.21
T130A	13.66	13.93	1.42	0.29	WT	13.45	13.72	1.40	0.12
E129A	13.99	14.41	1.15	0.23					

a) This data is also presented in visual form in Fig. 8. All energies are shown in kcal·mol^{-1}. All calculated values presented here are averages and standard deviations/standard error of the mean over 24 individual trajectories.

Table S4 Calculated activation free energies from the separate site-saturation mutagenesis of positions 93, 164 and 172.[a)]

Mutant	Average	Median	Standard deviation	Standard error of mean	Mutant	Average	Median	Standard deviation	Standard error of mean
L93A	11.83	11.79	1.23	0.49	T172N	13.43	13.55	1.17	0.47
L93C	13.26	13.00	0.95	0.38	T172P	14.44	14.20	0.75	0.30
L93D	14.07	13.79	1.02	0.41	T172Q	13.50	13.92	1.90	0.76
L93E	13.64	13.83	1.25	0.50	T172S	14.00	14.61	1.17	0.47
L93F	13.80	14.09	1.14	0.46	T172V	13.80	14.23	1.20	0.48
L93G	12.99	13.01	0.82	0.33	T172W	13.63	14.04	1.90	0.76
L93H	13.70	13.86	0.83	0.33	T172Y	13.43	13.70	1.39	0.55
L93I	13.58	13.61	1.04	0.42	Y164A	11.85	10.89	2.15	0.86
L93M	13.41	14.03	1.49	0.60	Y164C	10.82	10.24	1.44	0.58
L93N	14.64	14.81	1.40	0.56	Y164D	11.19	11.51	1.06	0.42
L93P	12.49	12.43	0.64	0.25	Y164E	10.58	10.29	1.48	0.59
L93S	13.30	13.56	1.32	0.53	Y164F	13.53	13.95	1.35	0.54
L93T	12.40	12.35	1.11	0.44	Y164G	13.23	14.00	2.19	0.87
L93V	13.20	13.94	1.85	0.74	Y164H	12.35	12.43	1.67	0.67
L93W	N.D. [b)]	N.D. [b)]			Y164I	13.63	13.47	1.05	0.42
L93Y	14.21	14.34	0.92	0.37	Y164L	13.21	14.03	1.94	0.78
T172A	13.23	13.76	1.49	0.60	Y164M	13.35	13.93	1.55	0.62
T172C	14.03	13.96	0.73	0.29	Y164N	12.84	12.67	1.62	0.65
T172D	13.04	13.14	1.95	0.78	Y164P	9.06	9.01	0.45	0.18
T172E	13.97	14.38	1.63	0.65	Y164Q	13.81	14.06	1.19	0.48
T172F	13.95	14.64	2.20	0.88	Y164S	12.04	11.56	2.56	1.02
T172G	11.61	11.54	1.59	0.64	Y164T	14.37	14.35	0.78	0.31
T172H	13.87	14.30	1.04	0.42	Y164V	13.58	14.39	2.11	0.84
T172I	13.68	13.79	0.87	0.35	Y164W	12.46	12.44	0.82	0.33
T172L	13.49	14.39	1.83	0.73	WT	13.45	13.72	1.40	0.12
T172M	13.43	13.13	1.09	0.44					

[a)] This data is also presented in visual form in Fig. 9. All energies are shown in kcal·mol^{-1}. All calculated values presented here are averages and standard deviations/standard error of the mean over 24 individual trajectories.

[b)] This reaction was calculated to have extreme endothermicity, causing the transition state to disappear from the calculated free energy profile. The free energy mapping failed for these results.

Table S5 Calculated activation free energies from the simultaneous partial saturation mutagenesis of positions 93, 164 and 172. [a)]

Position 164 replacement	Position 172 replacement	Average	Median	Standard deviation	Standard error of mean
Y164A	T172D	11.51	11.35	1.50	0.31
	T172L	11.70	11.05	2.28	0.46
	T172R	12.64	12.01	1.71	0.35
	T172S	10.28	10.01	1.07	0.22
	T172W	10.63	10.33	1.00	0.20
Y164C	T172D	13.36	14.10	2.30	0.47
	T172L	13.88	15.00	2.70	0.55
	T172R	11.34	10.87	1.96	0.40
	T172S	12.75	12.77	1.65	0.34
	T172W	11.39	11.13	2.20	0.45
Y164E	T172D	10.49	10.44	1.54	0.31
	T172L	10.48	10.28	1.61	0.33
	T172R	10.94	11.46	1.80	0.37
	T172S	10.54	10.35	1.99	0.41
	T172W	11.90	11.93	1.25	0.26
Y164H	T172D	14.67	14.58	1.55	0.32
	T172L	13.00	13.44	1.67	0.34
	T172R	13.07	13.36	0.96	0.20
	T172S	14.05	13.53	1.93	0.39
	T172W	13.19	13.84	1.67	0.34
Y164P	T172D	11.43	10.92	2.29	0.47
	T172L	9.75	9.38	1.06	0.22
	T172R	9.92	9.60	1.26	0.26
	T172S	10.50	9.23	2.43	0.50
	T172W	10.92	10.32	1.93	0.39
Y164S	T172D	10.99	10.76	1.42	0.29
	T172L	12.81	11.89	2.47	0.50
	T172R	12.74	12.11	1.91	0.39
	T172S	11.05	9.72	2.52	0.51
	T172W	11.42	10.34	2.11	0.43
WT	--	13.45	13.72	1.40	0.12

[a)] This data, which is split into Tables S5-S8 is visualized in Fig 10. All variants of this table show L93 at position 93. All energies are shown in kcal·mol^{-1}. All calculated values presented here are averages and standard deviations/standard error of the mean over 24 individual trajectories.

Table S6 Calculated activation free energies from the simultaneous partial saturation mutagenesis of positions 93, 164 and 172. [a)]

Position 164 replacement	Position 172 replacement	Average	Median	Standard deviation	Standard error of mean
Y164Y	T172D	14.16	14.17	1.33	0.27
	T172L	12.13	12.27	1.39	0.28
	T172R	12.11	11.90	0.71	0.15
	T172S	13.58	13.66	0.81	0.17
	T172W	12.35	12.42	1.55	0.32
Y164A	T172T	12.27	11.71	2.08	0.43
	T172D	11.04	10.52	1.65	0.34
	T172L	11.20	10.80	1.34	0.27
	T172R	10.01	9.87	1.04	0.21
	T172S	10.71	10.73	1.01	0.21
	T172W	10.13	9.85	1.00	0.20
Y164C	T172T	9.69	9.63	0.47	0.10
	T172D	12.79	13.48	1.89	0.39
	T172L	10.67	10.66	0.85	0.17
	T172R	11.41	10.14	2.39	0.49
	T172S	10.64	10.16	1.56	0.32
	T172W	10.90	10.95	1.12	0.23
Y164E	T172T	10.42	10.51	1.79	0.36
	T172D	10.42	10.15	1.42	0.29
	T172L	10.58	10.49	1.69	0.34
	T172R	9.15	8.90	1.28	0.26
	T172S	10.24	9.68	1.52	0.31
	T172W	9.61	9.48	1.22	0.25
Y164H	T172T	12.32	12.08	1.34	0.27
	T172D	13.14	13.00	0.84	0.17
	T172L	11.56	10.80	1.54	0.31
	T172R	12.08	12.02	0.67	0.14
	T172S	13.17	13.56	1.26	0.26
	T172W	13.32	13.35	1.02	0.21
Y164P	T172T	11.76	11.69	2.72	0.56
	T172D	11.09	11.13	1.10	0.22
	T172L	11.12	10.99	1.48	0.30
	T172R	12.32	12.46	1.51	0.31
	T172S	10.73	10.05	1.75	0.36
	T172W	10.95	10.10	1.70	0.35
Y164S	T172T	10.77	10.30	1.41	0.29
	T172D	12.84	11.43	2.96	0.60
	T172L	10.54	10.75	0.90	0.18
	T172R	9.87	9.65	1.27	0.26
	T172S	11.63	10.90	2.03	0.41
	T172W	11.01	10.97	1.45	0.30

[a)] This data, which is split into Tables S5-S8 is visualized in Fig 10. All variants shown in this table carry the L93A amino acid substitution. All energies are shown in kcal·mol^{-1}. All calculated values presented here are averages and standard deviations/standard error of the mean over 24 individual trajectories.

Table S7 Calculated activation free energies from the simultaneous partial saturation mutagenesis of positions 93, 164 and 172. [a)]

Position 164 replacement	Position 172 replacement	Average	Median	Standard deviation	Standard error of mean
Y164Y	T172D	12.96	12.73	2.02	0.41
	T172L	13.06	13.70	1.82	0.37
	T172R	13.90	14.17	1.20	0.25
	T172S	13.79	13.48	1.30	0.27
	T172W	13.97	14.37	1.32	0.27
Y164A	T172T	10.83	10.01	1.80	0.37
	T172D	10.38	10.22	1.03	0.21
	T172L	11.93	10.62	2.64	0.54
	T172R	12.37	13.01	2.13	0.43
	T172S	10.93	9.86	2.24	0.46
	T172W	9.82	9.85	0.70	0.14
Y164C	T172T	12.28	12.34	1.71	0.35
	T172D	13.11	12.95	2.30	0.47
	T172L	13.39	14.61	2.43	0.50
	T172R	10.89	10.24	2.18	0.44
	T172S	13.09	13.77	2.16	0.44
	T172W	13.11	13.37	1.59	0.33
Y164E	T172T	10.91	10.70	0.81	0.17
	T172D	11.41	10.56	2.47	0.50
	T172L	8.57	7.94	1.43	0.29
	T172R	9.93	9.44	1.74	0.35
	T172S	10.08	9.81	1.19	0.24
	T172W	10.40	10.04	1.29	0.26
Y164H	T172T	12.54	12.26	1.23	0.25
	T172D	13.14	13.66	1.55	0.32
	T172L	12.76	13.61	2.13	0.44
	T172R	12.31	12.55	1.47	0.30
	T172S	13.73	13.19	2.94	0.60
	T172W	13.90	13.98	0.57	0.12
Y164P	T172T	11.73	11.23	2.62	0.54
	T172D	12.34	12.02	2.49	0.51
	T172L	10.20	9.71	1.36	0.28
	T172R	11.34	10.94	2.04	0.42
	T172S	10.78	10.94	0.85	0.17
	T172W	9.85	9.80	0.89	0.18
Y164S	T172T	10.22	10.24	0.75	0.15
	T172D	10.72	10.52	1.52	0.31
	T172L	10.91	11.04	1.32	0.27
	T172R	11.89	11.14	2.78	0.57
	T172S	10.35	10.57	0.75	0.15
	T172W	12.13	11.45	2.55	0.52

[a)] This data, which is split into Tables S5-S8 is visualized in Fig 10. All variants of this table show G93 at position 93. All energies are shown in kcal·mol^{-1}. All calculated values presented here are averages and standard deviations/standard error of the mean over 24 individual trajectories.

Table S8 Calculated activation free energies from the simultaneous partial saturation mutagenesis of positions 93, 164 and 172. [a)]

Position 164 replacement	Position 172 replacement	Average	Median	Standard deviation	Standard error of mean
Y164Y	T172D	13.26	13.20	2.64	0.54
	T172L	13.73	13.65	0.99	0.20
	T172R	12.77	12.54	1.09	0.22
	T172S	13.39	13.50	0.89	0.18
	T172W	12.65	12.56	1.29	0.26
Y164A	T172T	12.09	11.39	2.40	0.49
	T172D	10.37	10.26	0.58	0.12
	T172L	12.91	13.61	1.51	0.31
	T172R	11.70	10.34	2.57	0.52
	T172S	12.39	12.28	2.04	0.42
	T172W	10.07	9.92	0.64	0.13
Y164C	T172T	10.96	10.48	1.70	0.35
	T172D	11.11	10.69	0.94	0.19
	T172L	13.70	13.75	2.00	0.41
	T172R	11.42	10.58	2.73	0.56
	T172S	11.81	11.64	1.20	0.24
	T172W	12.71	12.71	2.70	0.55
Y164E	T172T	11.50	10.88	1.40	0.29
	T172D	9.75	9.97	0.89	0.18
	T172L	10.29	10.15	1.22	0.25
	T172R	9.89	9.99	0.94	0.19
	T172S	10.02	10.15	1.79	0.36
	T172W	9.96	10.10	1.24	0.25
Y164H	T172T	13.37	13.59	1.05	0.21
	T172D	13.54	14.24	1.72	0.35
	T172L	13.40	14.09	1.52	0.31
	T172R	12.61	12.91	0.70	0.14
	T172S	12.86	13.46	1.55	0.32
	T172W	12.56	12.38	1.77	0.36
Y164P	T172T	9.98	9.45	1.30	0.27
	T172D	11.05	10.99	1.46	0.30
	T172L	13.38	13.37	1.62	0.33
	T172R	10.31	9.60	1.75	0.36
	T172S	11.06	11.22	0.88	0.18
	T172W	10.57	9.58	2.08	0.42
Y164S	T172T	13.17	13.50	2.03	0.41
	T172D	11.75	11.11	2.14	0.44
	T172L	10.68	10.82	0.87	0.18
	T172R	11.55	10.57	2.68	0.55
	T172S	12.47	12.13	1.20	0.25
	T172W	11.98	11.55	1.44	0.29

[a)] This data, which is split into Tables S5-S8 is visualized in Fig 10. All variants of this table show H93 at position 93. All energies are shown in kcal·mol^{-1}. All calculated values presented here are averages and standard deviations/standard error of the mean over 24 individual trajectories.

EVB Parameters

Table S9 EVB off-diagonal element (H_{ij}) and gas phase shift (α_i) parameters, obtained as described in the main text (see Section 2.1).

Substrate	H_{ij} (kcal·mol^{-1})	α_i (kcal·mol^{-1})
DHAP	60.0	229.0

Table S10 List of the atom types and Van der Waals parameters used for atoms constituting the reacting part of the system. For all atoms except reacting atoms, a standard 6-12 Lennard-Jones potential was used. In the case of the reacting atoms, which change bonding patterns between atoms *i* and *j*, an alternate function of the form $V_{react} = C_i C_j \, exp(-\alpha_I \alpha_j r_{ij})$ was used to prevent artificial repulsion between these atoms as bonding patterns change. r_{ij} denotes the distance (Å) between atoms *i* and *j*. For atom type assignment see Table S11.

Type	A_i (kcal$^{1/2}$· mol$^{1/2}$ Å^6)	B_i (kcal$^{1/2}$· mol$^{1/2}$ Å^3)	C_i (kcal· mol^{-1})	α_i (Å^2)	A_{1-4} (kcal$^{1/2}$· mol$^{1/2}$ Å^3)	B_{1-4} (kcal$^{1/2}$· mol$^{1/2}$ Å^3)	Mass (a.u.)
C2	1802.24	34.18	1	2.5	1274.38	24.17	12.01
CDH	1103.59	24.67	180	2.6	780.35	17.44	12.01
CT	944.52	22.03	91	2.5	667.88	15.58	12.01
HA	69.58	4.91	1	2.5	49.20	3.47	1.01
HC	84.57	5.41	5	2.5	59.80	3.83	1.01
HO	0.00	0.00	5	2.5	0.00	0.00	1.01
O	616.44	23.77	1	2.5	435.89	16.81	16.00
O1	616.44	23.77	1	2.5	435.89	16.81	16.00
O2	616.44	23.77	250	1.5	435.89	16.81	16.00
O2Z	873.90	27.96	1	2.5	617.94	19.77	16.00
ODE	601.15	22.27	250	1.5	425.08	15.74	16.00
ODH	976.93	31.26	1	2.5	690.79	22.10	16.00
OH	760.65	25.05	1	2.5	537.86	17.71	16.00
OP	445.13	18.25	1	2.5	314.76	12.91	16.00
P	2447.79	46.79	1	2.5	1730.85	33.09	30.97

Table S11 Atom types in the different VB states (as depicted in Fig. S2) of atoms constituting the reacting part of the system. See Fig. S4 for atom numbering, Table S10 for the corresponding Van der Waals parameters and Table S12 for the corresponding partial charges. For atom numbering see Fig. S4.

Atom number	State I	State II
1	P	P
2	O2Z	O2Z
3	O2Z	O2Z
4	O2Z	O2Z
5	OP	OP
6	CT	CT
7	HC	HC
8	HC	HC
9	C2	CDH
10	O	ODH
11	CT	CDH
12	HC	HO
13	HC	HA
14	OH	OH
15	HO	HO
16	CT	CT
17	HC	HC
18	HC	HC
19	C2	C2
20	O2	ODE
21	O2	O1

Table S12 Atomic partial charges in the different VB states (as depicted in Fig. S2). These were obtained according to the procedure described in Section 2.4.1 of the main text. For atom numbering see Fig. S11.

Atom number	State I	State II
1	1.3336	1.3059
2	-0.9265	-0.9666
3	-0.9265	-0.9666
4	-0.9265	-0.9666
5	-0.5731	-0.5056
6	0.2501	0.1700
7	-0.0386	-0.0255
8	-0.0386	-0.0255
9	0.5084	0.3517
10	-0.5961	-0.8006
11	0.3072	-0.4685
12	-0.0187	0.4500
13	-0.0187	0.1608
14	-0.7439	-0.6532
15	0.4079	0.3883
16	-0.2200	-0.1200
17	0.0600	0.0600
18	0.0600	0.0600
19	0.7000	0.5200
20	-0.8000	-0.5300
21	-0.8000	-0.4400

Table S13 Bond types and corresponding parameters for covalent bonds of the reacting part of the system. Bonds of non reacting atoms are described as harmonic potentials $V_{Harmonic} = 0.5k\ (r_{ij} - r_0)^2$, while bonds of reacting atoms are described as Morse potentials $V_{Morse} = De\ \{1 - \exp[-\alpha\ (r_{ij} - r_0)]\}^2$. For bond type assignment see Table S14.

Bond type	E_D (kcal·mol^{-1})	α (Å^{-2})	r_0 (Å)	k_b (kcal·mol^{-1}. Å^{-2})	b (Å)
0			Not Set		
1				1312	1.250
2				900	1.364
3				1140	1.229
4	245.8	1.5	0.945		
5				634	1.522
6				634	1.510
7				1140	1.229
8				900	1.370
9				1098	1.340
10	85.0	2.0	1.090		
11				680	1.090
12				680	1.080
13				640	1.410
14				900	1.370
15				1050	1.480
16				460	1.610
17				640	1.410
18				1106	0.945
19				536	1.529

Table S14 Bond type assignment for the covalent bonds of the reacting part of the system in the different VB states (as depicted in Fig. S2). For atom numbering see Fig. S4.

Atom number		Bond type	
#1	#2	State I	State II
1	2	15	15
1	3	15	15
1	4	15	15
1	5	16	16
5	6	17	17
6	9	5	6
6	7	11	11
6	8	11	11
9	10	7	8
9	11	5	9
11	12	10	0
11	13	11	12
11	14	13	14
14	15	18	18
16	17	11	11
16	18	11	11
16	19	5	5
19	20	1	2
19	21	1	3
20	12	0	4
R[a]	16	19	19

[a)] R corresponds to C_{β} in E165, which has no assigned atom number as it is not part of the reacting system (the corresponding PDB atom number is used in the setup of the simulations).

Table S15 Angle types and the corresponding parameters used for bending adjacent bonds in the reacting part of the system. The angle potential is described as $V_{angle} = 0.5\ \Sigma k(\Theta\text{-}\Theta_0)^2$. For the assignment of angle types see Table S16.

Angle type	k_a ($kcal{\cdot}mol^{-1}{\cdot}rad^{-2}$)	Θ (°)	Angle type	k_a ($kcal{\cdot}mol^{-1}{\cdot}rad^{-2}$)	Θ (°)
0	Not Set		13	100.0	109.50
1	140.0	117.00	14	140.0	123.00
2	160.0	120.40	15	140.0	123.00
3	70.0	113.00	16	110.0	108.50
4	100.0	109.50	17	70.0	113.00
5	140.0	120.00	18	66.0	107.80
6	160.0	120.40	19	70.0	109.50
7	140.0	123.00	20	70.0	114.50
8	140.0	116.00	21	200.0	108.23
9	140.0	124.00	22	200.0	120.50
10	70.0	109.50	23	70.0	109.50
11	70.0	109.50	24	75.0	110.70
12	70.0	120.00	25	126.0	111.10

Table S16 Angle type assignment in the different VB states (as depicted in Fig. S2). For atom numbering see Fig. S4.

Atom number			Angle type	
#1	#2	#3	State I	State II
1	5	6	22	22
5	6	9	4	5
5	1	2	21	21
5	1	3	21	21
5	1	4	21	21
5	6	7	23	23
5	6	8	23	23
6	9	10	6	7
6	9	11	8	9
7	6	9	10	11
8	6	9	10	11
9	11	12	10	0
9	11	13	10	12
9	11	14	13	14
10	9	11	6	15
11	14	15	16	17
12	11	13	18	0
12	11	14	19	0
13	11	14	19	20
16	19	21	1	2
17	16	19	10	10
18	16	19	10	10
19	20	12	0	3
R[a)]	16	17	24	24
R [a)]	16	18	24	24
R [a)]	16	19	25	25

[a)] R corresponds to C_{β} in E165, which has no assigned atom number as it is not part of the reacting system (the corresponding PDB atom number is used in the setup of the simulations).

Table S17 Torsion types and corresponding parameters used in the reacting part of the system. The torsion angle potential is described as $V_{torsion} = V_1(1+\cos(n\varphi-\delta)) + V_2(1+\cos2(n\varphi-\delta)) + V_3(1+\cos3(n\varphi-\delta))$, here *n* is the periodicity (number of maxima per turn) and δ is the phase shift. For torsion type assignment see Table S18.

Torsion type	V_1	V_2	V_3	Torsion type	V_1	V_2	V_3
	0.5.barrier height (kcal·mol^{-1})				0.5.barrier height (kcal·mol^{-1})		
0	Not Set			14	0.0000	7.0000	0.0000
1	0.0000	0.2730	0.0000	15	0.0000	0.0000	0.0000
2	0.0000	0.5830	0.0000	16	0.0000	0.0000	0.0000
3	1.5000	2.4500	0.0000	17	0.0000	0.0000	-0.1860
4	0.0000	0.0000	0.0000	18	-0.1780	-0.0870	0.2460
5	0.0000	0.0000	0.0000	19	0.7500	0.0000	0.0000
6	0.0000	2.4500	0.0000	20	0.0000	7.0000	0.0000
7	-0.2750	0.0000	0.0000	21	2.1590	0.0000	0.0000
8	2.1595	0.0000	0.0000	22	0.0000	7.0000	0.0000
9	0.8550	-0.2500	0.3310	23	0.0000	0.0000	0.2250
10	0.2500	0.0000	0.0000	24	0.0000	2.7500	0.0000
11	0.0000	0.0000	0.1370	25	0.0000	0.0000	0.2810
12	0.0000	7.0000	0.0000	26	0.0000	0.0000	0.1790
13	0.8550	-0.2500	0.3310	27	0.0000	1.4950	0.0000

Table S18 Torsion type assignment in the different VB states (as depicted in Fig. S2). For atom numbering see Fig. S4.

Atom number				Torsion type	
#1	#2	#3	#4	State I	State II
1	5	6	7	26	26
1	5	6	8	26	26
1	5	6	9	27	27
2	1	5	6	25	25
3	1	5	6	25	25
4	1	5	6	25	25
5	6	9	10	7	8
5	6	9	11	9	10
6	9	11	12	11	0
6	9	11	13	11	12
6	9	11	14	13	14
7	6	9	10	15	16
7	6	9	11	11	17
8	6	9	10	15	16
8	6	9	11	11	17
9	11	14	15	18	19
10	9	11	12	15	0
10	9	11	13	15	20
10	9	11	14	21	22
12	11	14	15	23	0
13	11	14	15	23	24
16	19	20	12	0	3
17	16	19	21	4	5
18	16	19	21	4	5
21	19	20	12	0	6
R [a)]	16	19	20	1	2
R[a)]	16	19	21	1	1

[a)] R corresponds to C_β in E165, which has no assigned atom number as it is not part of the reacting system (the corresponding PDB atom number is used in the setup of the simulations).

Table S19 Improper torsion types and corresponding parameters. The improper torsion potential is described as $V_{improper} = k\,(\tau - \tau_0)^2$, where k_i is the force constant and τ is the equilibrium angle (in degrees). For torsion type assignment see Table S20.

Improper type	k_a (kcal·mol^{-1} rad^{-2})	τ_0 (°)
0	Not set	
1	15.0	180
2	10.5	180
3	15.0	180

Table S20 Improper torsion type assignment in the different VB states (as depicted in Fig. S2). For atom numbering see Fig. S4.

Atom number				Improper type	
#1	#2	#3	#4	State I	State II
9	11	13	14	0	1
10	9	6	11	2	3
16	19	20	21	2	2

References

Blacklow, S. C. & Knowles, J. R. (1990) *Biochemistry* **29**, 4099-4108.

Malabanan, M. M., Amyes, T. L. & Richard, J. P. (2011) *J. Am. Chem. Soc.* **133**, 16428-16431.

Richard, J. P. (2012). *Biochemistry* **51**, 2652-2661.

Richard, J. P., Amyes, T. L., Malabanan, M. M., Zhai, X., Kim, K. J., Reinhardt, C. J., Wierenga, R. K., Drake, E. J. & Gulick, A. M. (2016) *Biochemistry* **55**, 3036-3047.

Zhai, X., Amyes, T. L. & Richard, J. P. (2015) *J. Am. Chem. Soc.* **137**, 15185-15197.

Zhai, X., Amyes, T. L., Wierenga, R. K., Loria, J. P. & Richard, J. P. (2013) *Biochemistry* **52**, 5928-5940.
